# Inactivation of the *dnaK* gene in *Clostridium difficile* 630 Δ*erm* yields a temperature-sensitive phenotype and increases biofilm-forming ability

**DOI:** 10.1038/s41598-017-17583-9

**Published:** 2017-12-13

**Authors:** Shailesh Jain, Deborah Smyth, Barry M. G. O’Hagan, John T. Heap, Geoff McMullan, Nigel P. Minton, Nigel G. Ternan

**Affiliations:** 1 Nutrition Innovation Centre for Food and HEalth (NICHE), School of Biomedical Sciences,University of Ulster, Coleraine, Co. Londonderry, N. Ireland, BT52 1SA UK; 2 Centre for Synthetic Biology and Innovation, Department of Life Sciences, Imperial College London, South Kensington Campus, London, SW7 2AZ UK; 3 Institute for Global Food Security, School of Biological Sciences, Queen’s University Belfast, N. Ireland, United Kingdom, BT9 7BL UK; 4 Clostridia Research Group, BBSRC/EPSRC Synthetic Biology Research Centre (SBRC), University of Nottingham, University Park, Nottingham, NG7 2RD UK

## Abstract

*Clostridium difficile* infection is a growing problem in healthcare settings worldwide and results in a considerable socioeconomic impact. New hypervirulent strains and acquisition of antibiotic resistance exacerbates pathogenesis; however, the survival strategy of *C. difficile* in the challenging gut environment still remains incompletely understood. We previously reported that clinically relevant heat-stress (37–41 °C) resulted in a classical heat-stress response with up-regulation of cellular chaperones. We used ClosTron to construct an insertional mutation in the *dnaK* gene of *C. difficile* 630 Δ*erm*. The *dnaK* mutant exhibited temperature sensitivity, grew more slowly than *C. difficile* 630 Δ*erm* and was less thermotolerant. Furthermore, the mutant was non-motile, had 4-fold lower expression of the *fliC* gene and lacked flagella on the cell surface. Mutant cells were some 50% longer than parental strain cells, and at optimal growth temperatures, they exhibited a 4-fold increase in the expression of class I chaperone genes including *GroEL* and *GroES*. Increased chaperone expression, in addition to the non-flagellated phenotype of the mutant, may account for the increased biofilm formation observed. Overall, the phenotype resulting from *dnaK* disruption is more akin to that observed in *Escherichia coli dnaK* mutants, rather than those in the Gram-positive model organism *Bacillus subtilis*.

## Introduction

*Clostridium difficile* is recognised as the most common cause of infectious antibiotic-associated bacterial diarrhoea in healthcare settings worldwide^1^. During dysbiosis in the gut, *C. difficile* infects human colonic epithelial cells, whereupon its toxins disrupt epithelial cell ultrastructure and thus the integrity of the gut epithelial barrier^2,3^. Symptoms include mild, self-limiting diarrhoea, cramping and low-grade fever (up to 40.6 °C); however, untreated *C. difficile* infection (CDI) can be life threatening^4^. Treatment generally comprises oral administration of antibiotics such as metronidazole, vancomycin or the recently introduced fidaxomycin^5,6^.

Cases of CDI have been exacerbated by the recent emergence of new, hypervirulent strains of the organism, and are associated with more severe infections, higher recurrence rates and higher mortality^7^. Antibiotic resistance plays an important role in driving these epidemiological changes, but despite extensive characterisation of the organism’s pathogenesis^8–11^, and its epidemiology and global spread^12–16^, the survival strategy of *C. difficile* in the challenging gut environment still remains incompletely understood^17,18^.

Genomic investigations have shown that, worldwide, a variety of lineages of *C. difficile* exist with differences in genome content^16,19,20^. Post-genomic comparative approaches have subsequently provided insights into genes, pathways and metabolic processes modulated under clinically relevant *in vitro* culture conditions (e.g. heat, antibiotics, oxygen, pH) or in *in vivo* models of CDI^21–28^. However, the precise function of clostridial genes has been difficult to determine considering the lack of genetic manipulation tools. Since the 1990s, techniques including physical and chemical mutagenesis^29^, homologous recombination, antisense RNA, mobile group II introns (ClosTron) and more recently, CRISPR-Cas9 genome editing tools have been deployed for ever more precise genome editing in clostridia^30,31^. The ClosTron, developed by Heap *et al*.^32,33^, utilises a retargeted mobile group II intron to allow targeted, permanent gene disruptions and the introduction of an erythromycin resistance gene, *ermB*, that enables positive selection of mutants. ClosTron disruption mutants generated for a variety of genes involved in infection, virulence and primary metabolism have allowed insights into their individual roles and their influence on the global physiology of the cell. The reader is referred to Kuehne and Minton^34^ for a comprehensive summary of the ClosTron technology, intron design procedures and mutant nomenclature.We previously demonstrated up-regulation of class I heat shock genes in *C. difficile* strain 630 in response to mild, clinically relevant heat-stress ranging from 37 °C to 41 °C^26–28^. Class I heat shock genes are members of the heat-inducible HrcA regulon and chaperone proteins encoded by the groESL and dnaJK–GrpE operons play pivotal roles in refolding denatured cellular proteins under stressful conditions such as pH (acid/alkali), O_2_ or antibiotic stresses^21,26–28^. To dissect the *C. difficile* heat-stress response in detail, we utilised ClosTron to attempt to create knockout mutants of the class I molecular chaperones *dnaK* and *groEL*, in addition to their negative transcriptional regulator *hrcA*.

## Results

We previously reported on the effects of clinically relevant heat-stress on the proteome and transcriptome of *C. difficile* strain 630, showing that a 4 °C temperature upshift (37–41 °C) resulted in a classical heat-stress response characterised by the up-regulation of various class I and III chaperones and cell-surface adhesins in addition to increased expression of Fe-only hydrogenases. A decrease in expression was noted for peptidyl prolyl *cis-trans* isomerases, *tcdA*, various cellular transport systems and certain motility-associated genes, including the flagellar gene *fliC* ^26–28^. In the current work, we hypothesised that disruption of key cellular chaperones would lead to pleiotropic changes in the physiology of *C. difficile*.

### ClosTron mutant construction

**Figure 1:**
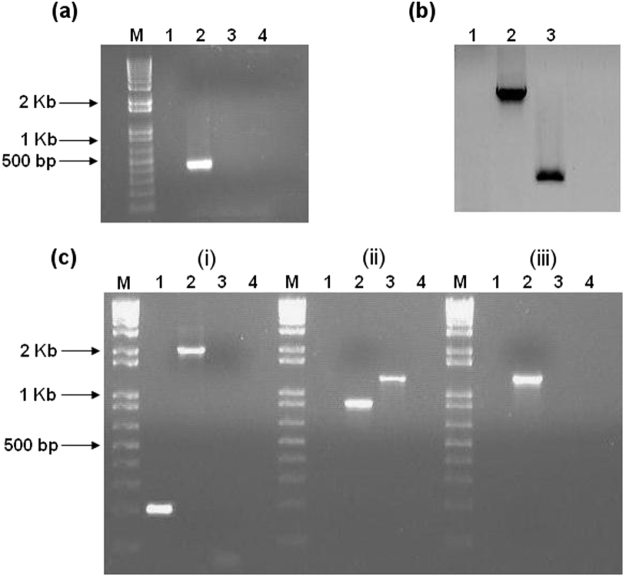
Validation of *C. difficile* 630 Δ*erm::dnaK* 723a mutant by PCR screening and Southern blotting. Lanes for each gel/experiment were loaded with PCR products as follows: M, 1 kb Plus DNA ladder (Invitrogen); Lane 1, *C. difficile* 630 Δ*erm*; Lane 2, *dnaK* mutant; Lane 3, pMTL007C-E2 plasmid DNA; Lane 4; negative control (water). **(a)** PCR across the intron-exon junction using EBS universal and Cdi-*dnaK*-R primers generated a 428 bp product from *dnaK* mutant (lane 2) showing presence of the intron; **(b**) Southern blot analysis to confirm single genomic insertion of the intron: An intron-specific probe for the ErmRAM was hybridised to *Hin*dIII-digested: genomic DNA extracted from *C. difficile* 630 Δ*erm* (Lane 1), pMTL007C-E2 plasmid DNA (Lane 2, positive control), and genomic DNA from the *dnaK* mutant (Lane 3). **(c)** Additional confirmatory PCR: **(i)** PCR using Cdi-*dnaK*-F and Cdi-*dnaK*-R primers generated a 210 bp product from *C. difficile* 630 Δ*erm* (lane 1), whereas the *dnaK* mutant produced a 2059 bp product, indicating the insertion of the group II intron (lane 2); **(ii)** PCR using ErmRAM-F and ErmRAM-R primers generated a 900 bp product from the *dnaK* mutant (lane 2) indicative of splicing out of the *td* group I intron, whereas unmodified pMTL007C-E2 template generated a 1300 bp product (lane 3), **(iii)** PCR across the other intron-exon junction using ErmRAM-R and Cdi-*dnaK*-F primers generated a 1300 bp product from the dnaK mutant only (lane 2). These experiments confirm insertion of the group II intron into the *C. difficile* 630 Δ*erm* chromosome at the desired site and in the correct orientation, resulting in *dnaK* inactivation.

For the *dnaK* gene (target site 722|723a; score 6.925), PCR screening of erythromycin-resistant colonies confirmed the generation of a ClosTron knockout mutant (Fig. 1a). Southern blot analysis (Fig. 1b) using an intron-specific probe for ErmRAM further verified the existence of a single copy of the insertion element. As expected, the probe did not hybridise to genomic DNA from the Δ*erm* strain, but hybridised as a single band to genomic DNA from the *dnaK* mutant strain, thus confirming the insertion of the group II intron into the desired target gene. The insertion site was verified by sequencing across intron–exon junctions (Supplementary Data S1), and confirmatory PCR of the ErmRAM region was also performed (Fig. 1c). In the case of *groEL* (target site 600|601 s; score 8.766) and *hrcA* (target site 285|286 s; score 7.971), genomic DNA from >100 erythromycin-resistant clones was PCR screened. Despite this, no gene-specific disruptions could be identified. Another intron insertion site was then chosen for *groEL* (target site 688|689a; score 6.18) and *hrcA* (target site 199|200a; score 4.19), and plasmids were ordered from DNA2.0, as per Heap *et al*.^33^. Despite multiple attempts and intensive PCR screening, it was not possible to isolate verifiable disruption mutants of either *groEL* or *hrcA* in *C. difficile* using the ClosTron system.

Other researchers have attributed the inability to recover ClosTron mutants to functional inefficiency of group II introns^35^; however, it is known that the integration frequency of retargeted introns varies over several orders of magnitude, and sometimes the integration frequency is too low to detect^32^. Whether HrcA and GroEL are essential in *C. difficile—*as reported for certain other bacteria^36^—remains unclear, but further attempts to isolate *groEL* or *hrcA* mutants were not pursued.

### Growth characteristics of the dnaK mutant

**Figure 2:**
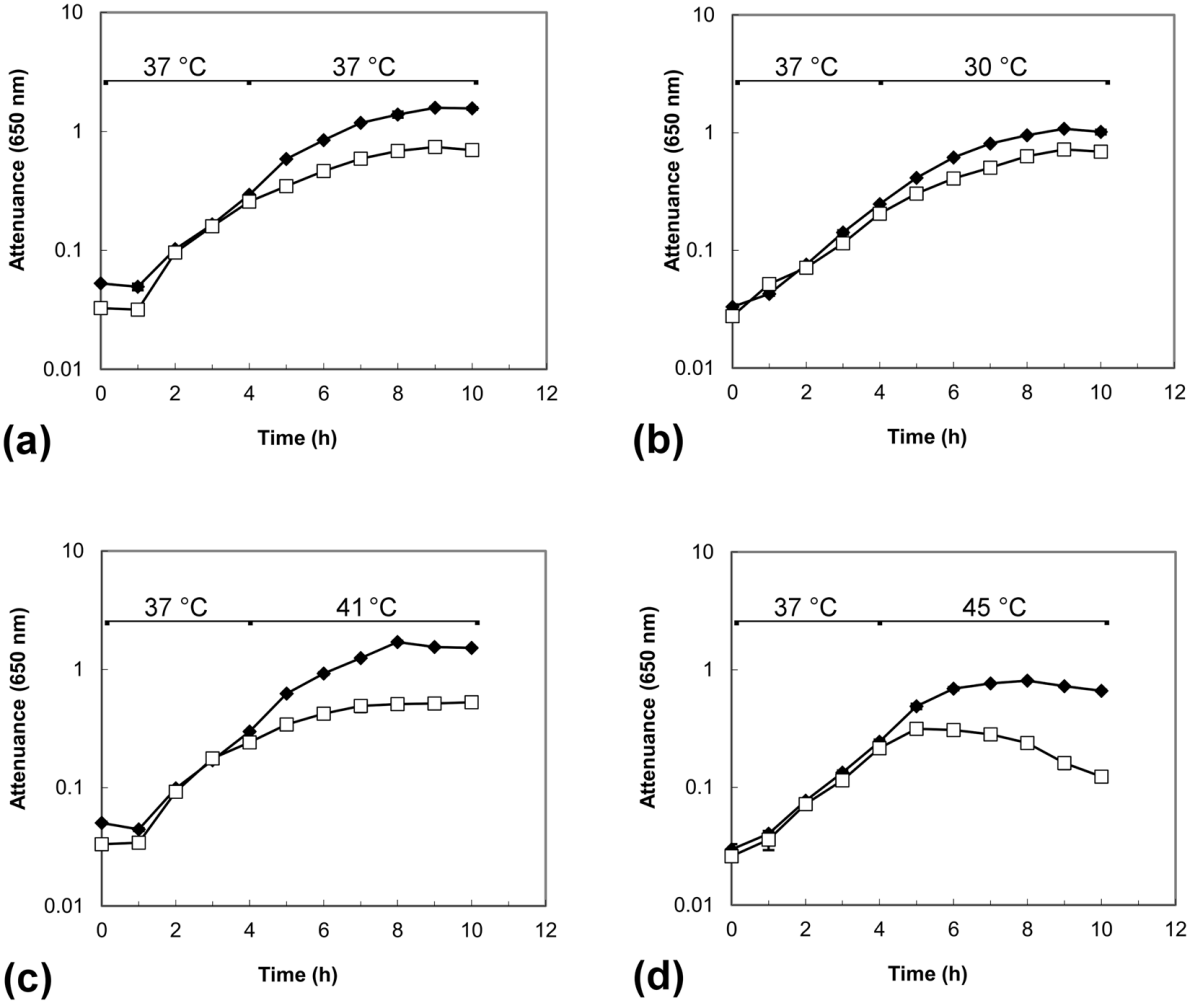
Growth of *C. difficile* 630Δ*erm* (◆) and *C. difficile* 630 Δ*erm::dnaK* 723a mutant (□) in BHIS broth at different temperatures. Temperature shifts were induced at early exponential phase, 4 h. **(a)** When grown at 37 °C, the *dnaK* mutant exhibited a temperature-sensitive phenotype, growing more slowly than *C. difficile* 630 Δ*erm*. **(b)** Cells grown to early exponential phase at 37 °C and then transferred to 30 °C grew in a comparable manner. Cells grown to early exponential phase at 37 °C were challenged by transfer to temperatures of **(c)** 41 °C and **(d)** 45 °C, respectively, where temperature sensitivity of the *dnaK* mutant was more pronounced. D_650nm_ values are plotted on a logarithmic scale and are averages of D_650nm_ measurements from biological triplicate cultures; error bars represent the standard error of mean.

Having isolated and verified the construction of *C. difficile* strain 630 *dnaK::Ll.ltrB-erm* (hereafter, the *dnaK* mutant), we investigated phenotypic changes compared to the Δ*erm* strain, initially considering growth rates and temperature sensitivity in BHIS broth. When grown at 37 °C (Fig. 2a), the *dnaK* mutant exhibited a temperature-sensitive phenotype, growing more slowly, and to a lower final attenuance, than *C. difficile* 630 Δ*erm*. In further experiments, cells were grown to early exponential phase (D_650nm_~0.3) at 37 °C, followed by transfer to 30 °C, 41 °C, or 45 °C. Upon transfer to 30 °C, both *C. difficile* 630 Δ*erm* and the *dnaK* mutant grew in a comparable manner (Fig. 2b). Raw attenuance data, with associated standard error of the mean values for these experiments can be found online in Supplementary Data S2. We previously determined using *C. difficile* strain 630 that there was no substantial difference in either growth rate or biomass production when the growth temperature was shifted from 37 °C to 41 °C^26^, indicating a certain robustness of this strain to temperature upshift. In the current work, however, we observed a considerable difference in the growth rate of the *dnaK* mutant as compared to the parental Δ*erm* strain following the induction of heat stress (Fig. 2c,d). This altered growth behaviour and thermosensitivity of the *dnaK* mutant could be interpreted as a direct consequence of *dnaK* inactivation.

### Disruption of dnaK results in impaired motility due to a *FliC*-deficient phenotype

**Figure 3:**
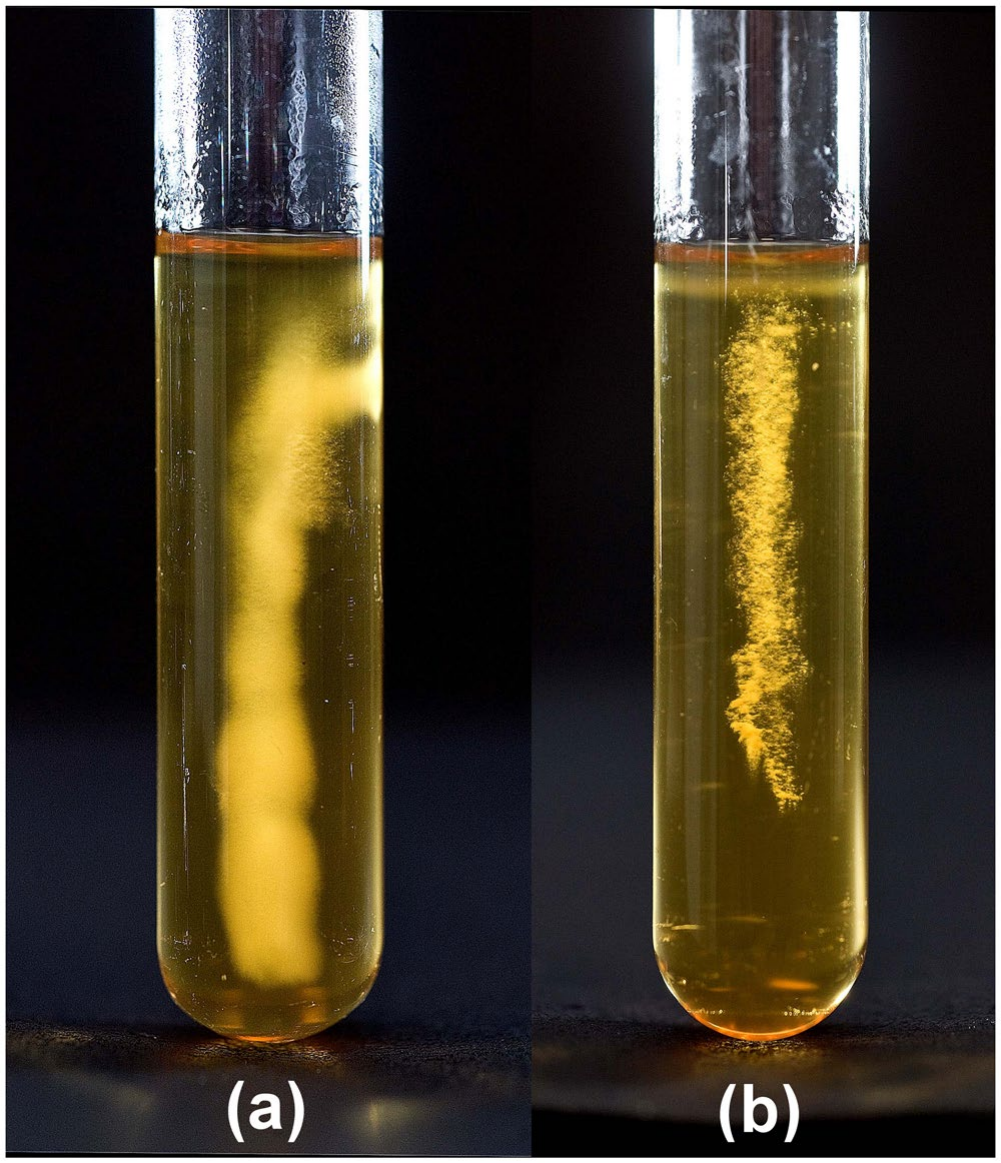
Motility of *C. difficile* strains in BHIS agar (0.175%). **(a)** *C. difficile* 630 Δ*erm*, **(b)** *dnaK* mutant. Motility was visualised as a diffuse spreading pattern from the point of stab inoculation.

**Figure 4:**
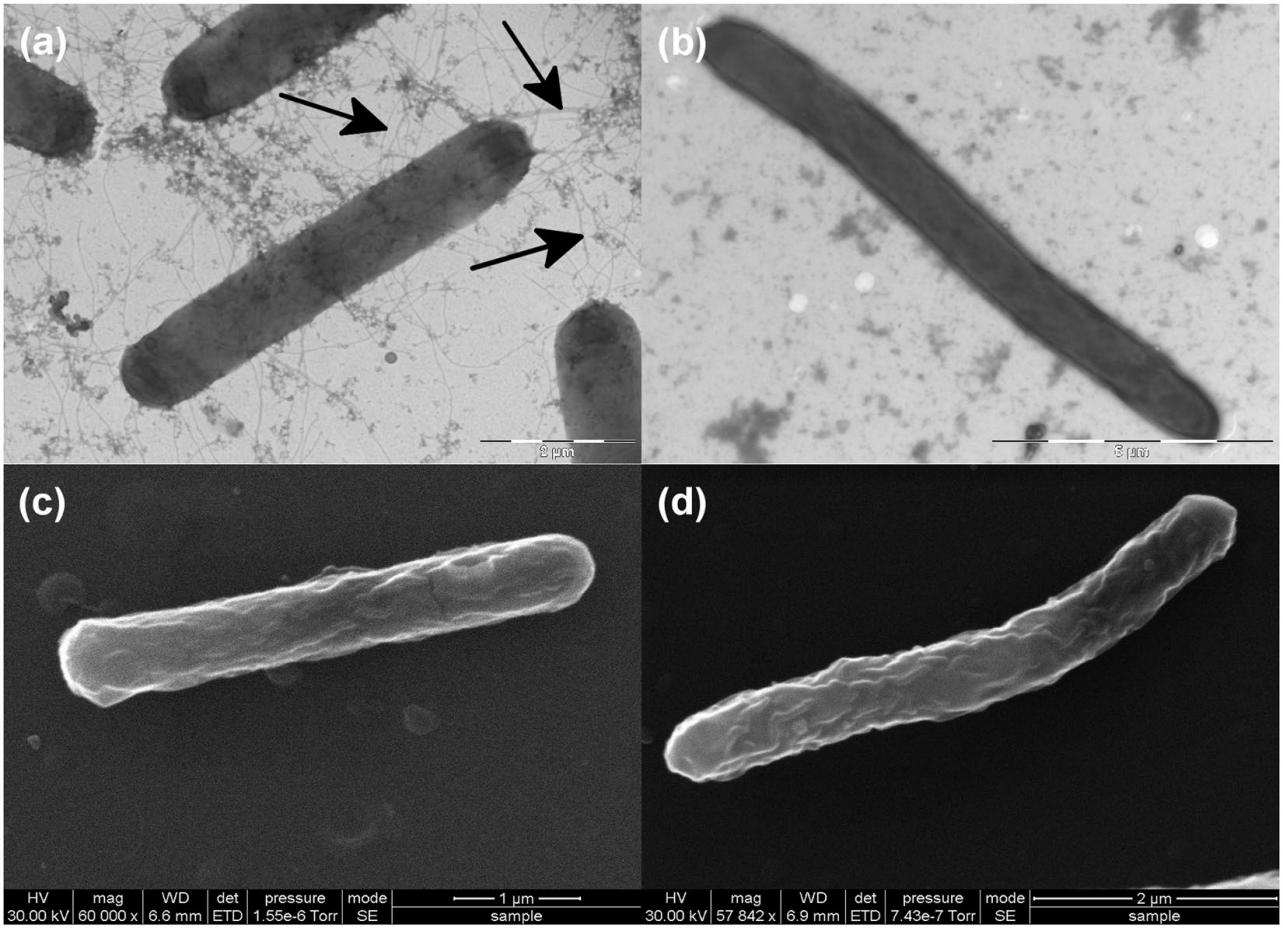
Electron microscopic analysis of *C. difficile* 630 Δ*erm* and *C. difficile* 630 Δ*erm*::*dnaK* 723a mutant. **(a)** Transmission electron microscopy image of *C. difficile* 630 Δ*erm*. **(b)** Transmission electron microscopy image of *dnaK* mutant. Arrows indicate flagellar filaments. **(c)** Scanning electron microscopy image of *C. difficile* 630 Δ*erm*. **(d)** Scanning electron microscopy image of *dnaK* mutant. The images depict the filamentous phenotype of the *dnaK* mutant in comparison to the wild-type.

The sensitivity of the *dnaK* mutant to elevated temperatures led to the hypothesis that a defect in DnaK function places the cells in a ‘heat-stress’ mode. This, we posited, would lead to a similar physiological response—including down regulation of *fliC*—to that observed in our earlier heat-stress experiments, where the expression of the gene encoding *fliC* was down-regulated. Thus, we assessed cellular motility by the method of Tasteyre *et al*.^37^ by stab inoculating *C. difficile* strains into motility agar tubes (in three replicates) and assessing growth following anaerobic incubation at 37 °C for 48 h. The parental *C. difficile* 630 Δ*erm* strain displayed a diffuse spreading pattern, with clear evidence of growth away from the inoculum stab, indicative of a motile phenotype (Fig. 3a). In contrast, the *dnaK* mutant (Fig. 3b) failed to produce the spreading pattern typical of motile organisms^37–39^. We hypothesised that this lowered motility could be due to the reduced expression of *fliC* or the lack of flagella on the *dnaK* mutant cell surface. This hypothesis was tested using both transmission and scanning electron microscopy (TEM and SEM, respectively) on cells grown at 37 °C. TEM with negative staining using phosphotungstic acid revealed that the parental *C. difficile* 630 Δ*erm* cells were peritrichously flagellated (Fig. 4a), while *dnaK* mutant cells did not have any visible flagella (Fig. 4b). This observation validated our hypothesis that the reduced motility of the *dnaK* mutant is attributable to the loss of the structural flagella machinery. TEM images indicated that *dnaK* disruption also resulted in a filamentous phenotype in the mutant (Fig. 4b), an observation further investigated using SEM, which clearly showed that cells of the *dnaK* mutant (Fig. 4d) were longer than those of the Δ*erm* strain [mutant cells, 9.04 ± 1.42 µm in length; wild-type cells, 6.72 ± 1.28 µm in length; 12 cells of each strain were measured]. In general, both wild-type and mutant cells also appeared slightly wrinkled, a phenomenon that can be attributed to the acetone dehydration step during the critical-point drying (CPD) process prior to SEM.

### Chaperone genes and fliC are differentially expressed in the dnaK mutant

**Figure 5:**
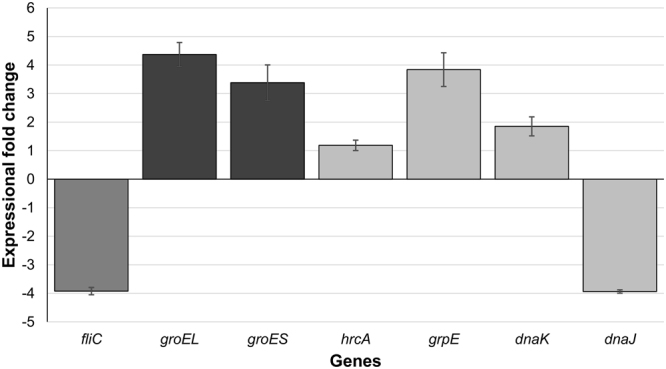
Expressional changes in class 1 chaperone genes and the flagellar filament gene, *fliC*, in the *C. difficile* 630 Δ*erm*::*dnaK* 723a mutant. RNA was extracted and reverse transcribed from biological duplicate cultures and cDNA was quantified in technical triplicate qPCR reactions. The ‘calibrator normalised relative quantification including efficiency correction’ experimental mode assessed gene expression using the *tpi* gene, whose expression did not change, as a reference. Bars represent average fold-changes in gene expression in the *dnaK* mutant compared with the Δ*erm* parental strain. Error bars represent standard deviation of the mean.

The lack of both motility and observable flagella, combined with the temperature sensitivity exhibited by the *dnaK* mutant, led us to the hypothesis that genes associated with these processes would be altered in the mutant. If, as we hypothesised, the *dnaK* mutant was in the ‘heat-stress mode’, then it was to be expected that expression of other chaperones would be increased as well. We also hypothesised that the lack of observable flagella could be underpinned by a decrease in *fliC* expression. Independent biological duplicate cultures of *C. difficile* 630 Δ*erm* and the *dnaK* mutant were grown at 37 °C and total RNA was isolated from cells harvested at the late-log phase, reverse transcribed to cDNA and the relative expression of chaperone genes and *fliC* was analysed with *tpi* as reference (see Supplementary Data S3 for ratios). Expression of *groEL*, *groES*, and *grpE* was increased in the *dnaK* mutant, whereas *dnaJ* expression decreased by more than 4-fold (Fig. 5). This is in broad accordance with what has been reported by other researchers, where increases in transcription of heat shock genes, compared to wild-type strains, were observed in *dnaK* null mutants grown at optimal growth temp eratures^40–42^. In addition, expression of *fliC* was 4-fold lower in the *dnaK* mutant, confirming that lower level of *fliC* transcript, as opposed to some defect in either translation or in the export of FliC monomers, was the primary reason for lack of flagellar motility.

### The dnaK mutant exhibits an increased biofilm-forming phenotype

**Figure 6:**
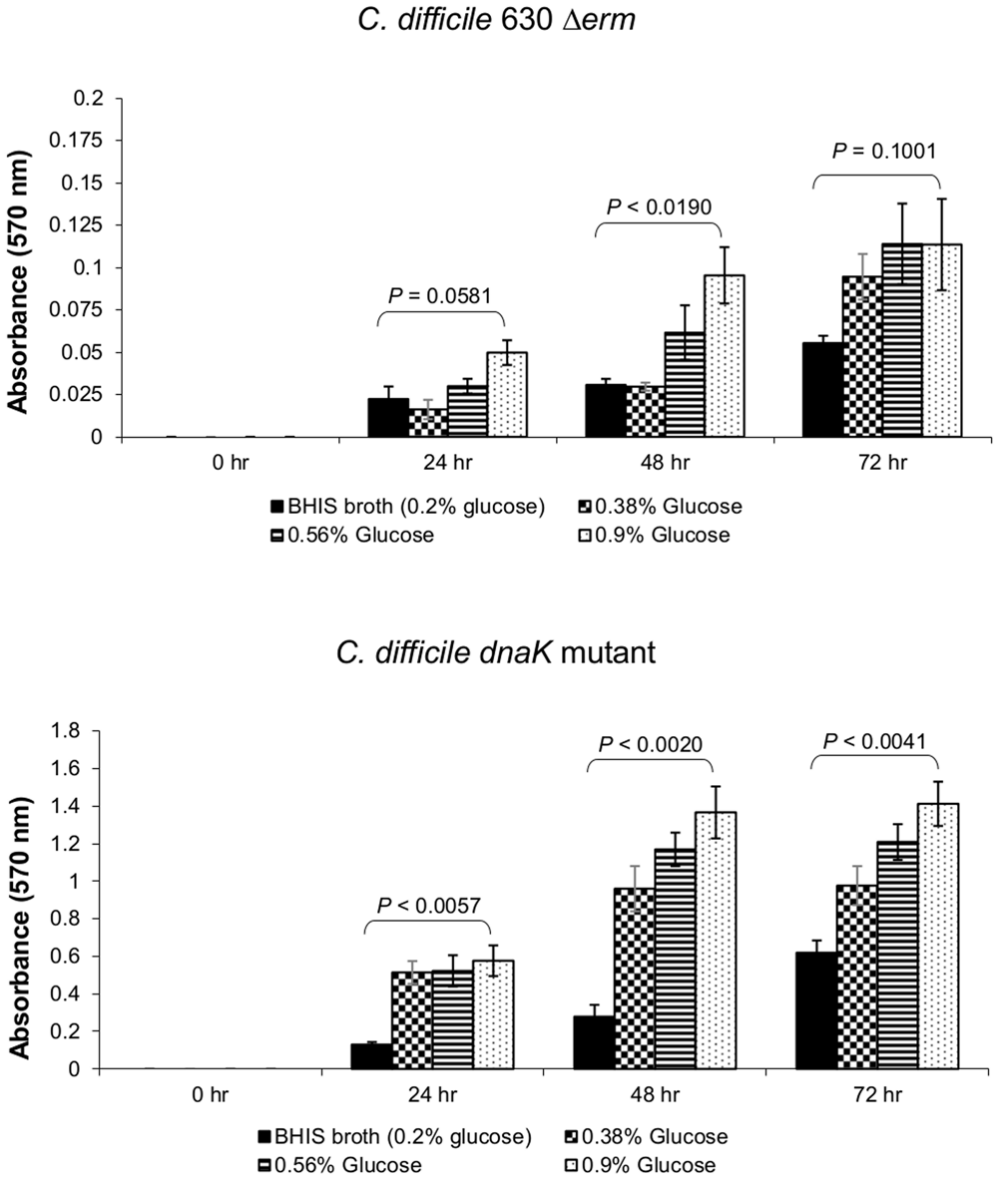
Biofilm-forming ability of *C. difficile* 630 Δ*erm* and *C. difficile* 630 Δ*erm*::*dnaK* 723a mutant. Biofilm assays were performed in biological triplicates, each with 6 independent technical replicates. Strains were classified as strong- (*A*_570_ > 1), moderate- (*A*_570_ = 0.5−1), or weak- (*A*_570_ < 0.5) biofilm producers^45^. P values represent statistical comparison (Student’s *t*-test, 2 tailed) between BHIS broth and BHIS broth with 0.9% (w/v) additional glucose.

In many bacteria, including *Pseudomonas aeruginosa*, both flagella and type IV pili contribute to motility and biofilm-forming ability^43^. We therefore assessed the ability of *C. difficile* strains to form biofilms using a modified version of the method of O’Toole and Kolter^44^, with the expectation that the flagella-deficient *dnaK* mutant would exhibit reduced biofilm-forming ability. To assess biofilm development, assays were performed in 96-well polystyrene microtiter plates (Orange Scientific, Alpha Technologies, UK) with measurements at 24, 48 and 72 h (see Supplementary Data S4). We observed that the *C. difficile* 630 Δ*erm* strain formed weak biofilms (*A*_570_ < 0.5, per the classification of Varga *et al*.^45^) (Fig. 6). In contrast, the *dnaK* mutant formed moderate to strong biofilms, and this ability was significantly enhanced at 24 h (p = 0.0057), 48 h (p = 0.0020) and 72 h (p = 0.0041) by the addition of 0.9% glucose to the BHIS broth (Fig. 6). By contrast, the effect of 0.9% glucose addition on biofilm production by *C. difficile* 630 Δ*erm* was significant only at 48 h (p = 0.0190), although biofilm biomass was increased at 24 h and 72 h compared to the BHIS control.

## Discussion

Here we report for the first time the successful construction, validation and phenotypic characterisation of a *C. difficile dnaK* disruption mutant using ClosTron. Our observations of impaired growth rates and lowered temperature stress tolerance with the *dnaK* mutant correspond with those of Selby *et al*.^46^ who made *dnaK* and *hrcA* mutants in *C. botulinum*. The *C. difficile dnaK* mutant had a lower growth rate and produced less biomass at temperatures between 30 °C and 45 °C (Fig. 2) and in addition was also less able to tolerate heat stress (Fig. 2), emphasising the importance of the DnaK chaperone system in protein folding, especially in relation to core cellular housekeeping functions. The results obtained using these two clostridial strains correspond with observations in other bacteria. In *Escherichia coli*, *dnaK* mutants reportedly grow more slowly and exhibit lower viability than the wild-type, exhibiting severe defects in DNA and RNA synthesis that account for the inhibited growth and reduced viability^47–49^. Accordingly, we noted that following lethal stress (2 min at 64 °C) and plating on BHIS agar, no *C. difficile dnaK* mutant cells were recovered, suggesting that *dnaK* mutation is deleterious to sporulation. We subsequently verified the presence of spores in cultures of the *C. difficile dnaK* mutant, but nonetheless, this markedly altered thermotolerance further emphasises the requirement for a functional DnaK chaperone in *C. difficile* physiology. Heat sensitivity has also been reported in *Lactococcus lactis* ^41^ and *Staphylococcus aureus* ^50^ *dnaK* mutants, yet in *Bacillus subtilis*, *dnaK* mutants were thermotolerant and could grow up to 52 °C, in addition to exhibiting a filamentous morphology^51^. Our motility experiments (Fig. 3) showed that the *C. difficile dnaK* mutant was less motile than the parental Δ*erm* strain. Electron microscopy revealed for the first time in *C. difficile* that the *C. difficile dnaK* mutant had no flagella (Fig. 4b) and that the mutant cells were approximately 50% longer than the parental Δ*erm* cells (Fig. 4d). This filamentous phenotype, reported in several other bacterial *dnaK* mutants^31,35,52^, could be due to either the accumulation of mutant DnaK proteins^52^ or some as yet unidentified defect in the expression/function of FtsZ, a highly conserved prokaryotic cytoskeleton protein which is the first protein to localise to the site of bacterial cell division and in turn defines the plane of cell division^53^. Sugimoto *et al*.^54^ reported that an *E. coli dnaK* deletion mutant displayed a filamentous morphology that was attributable to increased GrpE abundance which in turn interfered with DnaK chaperone system functionality: the imbalance resulted in defective cell division mediated via abnormal FtsZ localisation. The *C. difficile dnaK* mutant exhibited 3- to 4-fold increases in the expression of all class I heat shock genes (Fig. 5), with the exception of *dnaJ*, the expressions of which was 4-fold lower. We therefore indicate that intracellular concentrations of the molecular chaperones encoded by the *dnaK* operon may directly influence the activity and localisation of FtsZ (encoded by CD2646) in *C. difficile*. The reduction in *dnaJ* expression in the *dnaK* mutant is similar to that observed in *E. coli* ^49^ and could be due to either differential transcription or transcriptional attenuation. Our previous work reported differential expression of *dnaK* operon transcripts and proteins in *C. difficile* cells^26–28^. In *B. subtilis*, both transcriptional and post-transcriptional controls adjust cellular quantities of proteins derived from the *dnaK* operon, and a strategy of differential segmental mRNA stability is in place to fine-tune the expression of individual *dnaK* operon genes. The *B. subtilis dnaK* operon transcripts contain stemloop structures that act as rho-independent transcription terminators with one of these predicted upstream of *dnaJ*, and there is also a vegetative promoter just upstream of *dnaJ* in both *Bacillus* and *Clostridium* that is not regulated by heat stress^55,56^. Consequently, *dnaK* disruption may have wider effects on *C. difficile* transcription factors or mRNA processing. The possibility of *dnaK* disruption also giving rise to a polar effect on *dnaJ* cannot be discounted. While the dnaK ORF is disrupted and thus must be non-functional, a polar effect on the expression of neighbouring genes–including *dnaJ*–could be an alternative explanation for the observed decrease in *dnaJ* expression. In *Salmonella enterica* Serovar Typhimurium an insertion in the *dnaK* gene led to depletion of both DnaK and DnaJ^57^ and thus it could be argued that there may be a *dnaJ* polar effect component to the observed phenotype in *C. difficile*. The *C. difficile dnaK* mutant exhibited 3- to 4-fold increased expression of the *groESL* operon at 37 °C. A number of possibilities could explain this. In *E. coli*, the DnaK chaperone system is involved in the negative regulation of heat shock response by controlling the synthesis and stability of σ^32^, the positive regulator^42^. The absence of a functional DnaK protein leads to σ^32^ overproduction and thus *E. coli dnaK* mutants exhibit increased expression of molecular chaperones even at optimal growth temperatures^40^. This is what we observed in case of the *C. difficile dnaK* mutant. However, the regulation of heat shock response in Gram-positive microorganisms such as *B. subtilis* is much more complex, as multiple classes of heat shock genes have been identified^58^. Transcription of class I heat-inducible genes encoded by the *groE* and *dnaK* operon genes is negatively regulated by the HrcA repressor protein in conjunction with the CIRCE element, a palindromic sequence present in the promoter region of these operons^59,60^. In *B. subtilis*, GroEL is required for the stabilisation of HrcA, which in turn binds to the CIRCE element, blocking the transcription of class I heat-inducible genes at normal growth temperatures^61^. During stress, accumulation of unfolded proteins sequesters the activity of GroEL, causing inactivation of HrcA and allowing active transcription of the *groE* and *dnaK* operons. Thus, *dnaK* inactivation in *B. subtilis* does not result in an abnormal expression of class I heat shock proteins^51,62^. If the same mode of regulation holds for *C. difficile*, then *dnaK* disruption would not be expected to result in the overexpression of the *groESL*/*dnaK* operons. The *C. botulinum hrcA* mutant^46^ was reported to overexpress all six class I heat shock genes, as would be expected. Our observation that expression of the *groESL* operon was 4-fold higher in the *C. difficile dnaK* mutant suggests that in this organism, DnaK, rather than GroES/GroEL, might have a role to play in the stabilisation of HrcA and thus in the correct regulation of class I heat shock operons. There is clearly considerable diversity in the regulation networks and physiological roles of *dnaK* in different organisms, and regardless of the reasons for altered gene expression, the temperature-sensitive phenotype of the *C. difficile dnaK* mutant suggests that the protein folding defect resulting from *dnaK* disruption is only partially restored by subsequent increases in GroEL/GroES.

In *C. difficile*, the flagellum is an accessory virulence factor that promotes adherence to colonic epithelial cells at a level 10-fold higher than that of non-flagellated strains^37,38,63^. In the current work, the *C. difficile dnaK* mutant strain was non-motile, lacked surface flagella and *fliC* mRNA expression was 4-fold lower than that in the parental Δ*erm* strain. These observations are consistent with those reported for *B. subtilis* ^51^ and *E. coli* ^64^ where *dnaK* inactivation also resulted in a non-motile phenotype. It could be hypothesised that non-flagellated *C. difficile* cells would adhere weakly and thus be less virulent. We used a biofilm assay model to test adherence *in vitro* and showed that the *dnaK* mutant formed much more biofilm than the parental Δ*erm* strain (Fig. 6). This observation initially appears to be at odds with the literature consensus, that cell surface structure-driven motility is a vital factor in biofilm formation^43–45,65,66^. However, Hennequin *et al*.^67^ demonstrated that upon exposure of *C. difficile* to heat-shock conditions, GroEL was released into the extracytoplasmic space and became cell-surface adsorbed. They also provided evidence that in *C. difficile*, GroEL plays a role in adhesion, and further proposed that GroEL was, by default, associated with the membrane because of its chaperone activities^67^. The increased GroEL expression in the *C. difficile dnaK* mutant and this protein’s known role as an adhesin in *C. difficile* and also in other organisms such as *Salmonella typhimurium* ^68^, *Helicobacter* pylori^69^ and *Lactobacillus johnsonii* ^70^ suggest that increased GroEL expression is at least one of the factors responsible for the increased adherence and biofilm formation by the *dnaK* mutant.

To summarise, this paper reports for the first time the construction and characterisation of a ClosTron *dnaK* mutant in *C. difficile*. Our phenotypic characterisation clearly demonstrates that while DnaK is not essential for the viability of the organism, defects in DnaK functionality lead to altered expression of class I heat shock and motility genes, perturbations to the cell surface and adhesion and considerable disruption of global cellular physiology and homeostasis.

## Materials and Methods

### Bacterial strains and growth conditions

Bacterial strains and plasmids used in this work are listed in Table 1. *C. difficile* strains were anaerobically grown on BHIS agar or broth, as previously described^71^. For heat-stress experiments, liquid cultures growing at 37 °C were transferred to a recirculating 41 °C water-bath set at the appropriate temperature, as per Jain *et al*.^26^. *C. difficile* 630 Δ*erm* ^72^ was employed to allow selection of ClosTron mutants, *E. coli* TOP10 was used as the cloning host and *E. coli* CA434^73^ strain was the donor for conjugative transfer of plasmids to *C. difficile* 630 Δ*erm*.

**Table 1:** Strains/Plasmids used in this work.

Strain or Plasmid	Description	Source/Reference
**Strains**		
CD630	Wild-type (WT) strain	ATCC BAA-1382
CD630 Δ*erm*	Erm sensitive WT strain	Hussein *et al*.^72^
CD630 Δ*erm*::*dnaK* 723a	Strain with insertional inactivation of *dnaK*	This work
*E. coli* TOP10	Electrocompetent cloning strain	Invitrogen
*E. coli* CA434	Conjugation donor strain	Heap *et al*.^73^
**Plasmids**		
pMTL007-CE2	ClosTron mutagenesis vector	Heap *et al*.^33^
pMTL007-CE2::dnaK-722|723a score 6.925	ClosTron mutagenesis vector, intron retargeted to *dnaK*	This work
pMTL007-CE2::hrcA-285|286s score 7.971	ClosTron mutagenesis vector, intron retargeted to *hrcA*	This work
pMTL007-CE2::hrcA-199|200a score 4.19	ClosTron mutagenesis vector, intron retargeted to *hrcA*	This work
pMTL007-CE2::GroeL-600|601s score 8.766	ClosTron mutagenesis vector, intron retargeted to *groEL*	This work
pMTL007-CE2::groEL-688|689a score 6.18	ClosTron mutagenesis vector, intron retargeted to *groEL*	This work

### ClosTron mutagenesis procedure

*C. difficile* 630 Δ*erm* ^72^ was used and potential intron target sites in genes of interest were identified using the intron design tool available at http://clostron.com. Intron fragments for target genes were amplified by splicing overlap of extension (SOE) PCR, *Hin*dIII and *Bsr*GI digested and then ligated into the ClosTron plasmid pMTL007C-E2^33^. The construct was electroporated into *E. coli* TOP10 and transformants selected on LB agar with 25 µg/ml chloramphenicol and 8 µg/ml Xgal. The PCR product-derived portion of the retargeted plasmid was verified by sequencing with cspfdx-seq-F1 and pMTL007-R1 primers (Table 2). Retargeted, sequence-verified ClosTron plasmids for *dnaK, groEL* and *hrcA* were retransformed into electrocompetent *E. coli* CA434 cells, and the transformation mixtures were then used to inoculate 5 ml of sterile LB broth supplemented with 12.5 µg/ml chloramphenicol. Following overnight incubation (37 °C, 200 rpm), cells from 1 ml of culture were collected by centrifugation at 4 °C, washed in 0.5 ml sterile PBS and resuspended in 200 µl of an overnight culture of *C. difficile* 630 Δ*erm*. The entire conjugation mixture was pipetted onto fresh BHIS agar as a discrete drop and plates were anaerobically incubated for 8–10 h at 37 °C to allow conjugal transfer of the retargeted pMTL007C-E2 plasmid from *E. coli* CA434 to *C. difficile* 630 Δ*erm*.

**Table 2:** Oligonucleotides used in this work.

Strain or Plasmid	Description
Intron retargeting*	
Cdi-*dnaK*-722a -IBS	AAAAAAGCTTATAATTATCCTTAAATTCCTTCTTAGTGCGCCCAGATAGGGTG
Cdi-*dnaK*-722a -EBS1d	CAGATTGTACAAATGTGGTGATAACAGATAAGTCTTCTTAGCTAACTTACCTTTCTTTGT
Cdi-*dnaK*-722a -EBS2	TGAACGCAAGTTTCTAATTTCGATTGAATTTCGATAGAGGAAAGTGTCT
Cdi-*hrcA*-285s -IBS	AAAAAAGCTTATAATTATCCTTACTTATCGAACAAGTGCGCCCAGATAGGGTG
Cdi-*hrcA*-285s -EBS1d	CAGATTGTACAAATGTGGTGATAACAGATAAGTCGAACAATGTAACTTACCTTTCTTTGT
Cdi-*hrcA*-285s -EBS2	TGAACGCAAGTTTCTAATTTCGATTATAAGTCGATAGAGGAAAGTGTCT
Cdi-*hrcA*-200a -IBS	AAAAAAGCTTATAATTATCCTTACTTTTCCAGATGGTGCGCCCAGATAGGGTG
Cdi-*hrcA*-200a -EBS1d	CAGATTGTACAAATGTGGTGATAACAGATAAGTCCAGATGGATAACTTACCTTTCTTTGT
Cdi-*hrcA*-200a -EBS2	TGAACGCAAGTTTCTAATTTCGGTTAAAAGTCGATAGAGGAAAGTGTCT
Cdi-*groEL*-601s -IBS	AAAAAAGCTTATAATTATCCTTATTTGTCTCTGCAGTGCGCCCAGATAGGGTG
Cdi-*groEL*-601s -EBS1d	CAGATTGTACAAATGTGGTGATAACAGATAAGTCTCTGCATATAACTTACCTTTCTTTGT
Cdi-*groEL*-601s -EBS2	TGAACGCAAGTTTCTAATTTCGATTACAAATCGATAGAGGAAAGTGTCT
Cdi-*groEL*-689a -IBS	AAAAAAGCTTATAATTATCCTTACTGGTCATAATTGTGCGCCCAGATAGGGTG
Cdi-*groEL*-689a -EBS1d	CAGATTGTACAAATGTGGTGATAACAGATAAGTCATAATTCTTAACTTACCTTTCTTTGT
Cdi-*groEL*-689a -EBS2	TGAACGCAAGTTTCTAATTTCGGTTACCAGTCGATAGAGGAAAGTGTCT
EBS universal	CGAAATTAGAAACTTGCGTTCAGTAAAC
**ClosTron sequencing**	
cspfdx-seq-F1	GATGTAGATAGGATAATAGAATCCATAGAAAATATAGG
pMTL007-R1	AGGGTATCCCCAGTTAGTGTTAAGTCTTGG
**Screening of clones**	
Cdi-*dnaK*-F	CTACAGCTGGTAACAATAGATTAGGT
Cdi-*dnaK*-R	CTGTAGCAGTTATGAAAGGTAAGTT
Cdi-*groEL*-F	AGTCTCAAACTATGAATACTGAATTAGATG
Cdi-*groEL*-R	GCTTTTTACCTTGTTGAACTATTTGT
Cdi-*hrcA*-F	TAGGGTATTTAATTCAGCCTCATACTTC
Cdi-*hrcA*-R	TGCTACAGTTGTATAGTTTGTTAGTTGC
ErmRAM-F	ACGCGTTATATTGATAAAAATAATAATAGTGGG
ErmRAM-R	ACGCGTGCGACTCATAGAATTATTTCCTCCCG

*Introns were inserted after the indicated number of bases in the sense (s) or the antisense (a) orientation from the start of the open reading frame (ORF) of the target gene. Cdi, *C. difficile*; IBS, intron-binding sites; EBS, exon-binding sites; ErmRAM, erythromycin retrotransposition-activated selectable marker.

Following incubation, the mating mixture was recovered from the conjugation plates using a sterile loop and resuspended in 1 ml sterile PBS. To counter-select against *E. coli*, 200 µl of this conjugation slurry was spread onto fresh BHIS agar supplemented with *C. difficile* selective supplement (250 µg/ml of D-cycloserine and 8 µg/ml of cefoxitin; Oxoid), in addition to 15 µg/ml thiamphenicol to select for the retargeted pMTL007C-E2 plasmid. Following anaerobic incubation at 37 °C for 24–72 h, single, isolated, thiamphenicol-resistant colonies were re-streaked and grown on the same medium for a further 24 h. Integrants were then isolated by resuspending thiamphenicol-resistant colonies in 300 µl sterile PBS and plating (100 µl neat and 100 µl of 10-fold diluted) onto BHIS agar supplemented with 10 µg/ml of erythromycin to select for the presence of the spliced erythromycin retrotransposition-activated selectable marker (ErmRAM). Following anaerobic incubation at 37 °C for 24–72 h, erythromycin-resistant integrant colonies were re-streaked on the same medium for 24 h to ensure purity. A few integrant colonies were also replica plated onto BHIS-thiamphenicol (15 µg/ml) agar to screen for plasmid loss by the thiamphenicol-sensitive phenotype. Several erythromycin-resistant, thiamphenicol-sensitive clones were subsequently used to inoculate 1 ml of sterile BHIS broth, and cultures were incubated anaerobically at 37 °C overnight.

### Screening PCR

To confirm the generation of ClosTron mutants, the correct position of the intron in *C. difficile* mutant genomes was verified by PCR to determine whether the retargeted plasmid was present, whether the ClosTron had integrated and, more importantly, whether integration had taken place into the desired target gene in the *C. difficile* 630 Δ*erm* genome. Initial screening of mutants involved forward and reverse primers for the respective genes (Table 2) designed to yield ~200 bp amplicons from wild-type *dnaK*, *groEL* and *hrcA* genes and a larger ~2 kbp amplicon from ClosTron mutants due to intron insertion in these genes. Additional PCRs across the intron–exon junctions and to demonstrate a spliced—and therefore integrated—RAM were also performed. Screening PCRs used genomic DNA of wild-type *C. difficile* 630 Δ*erm* as a positive control, retargeted plasmid DNA to demonstrate a full-length RAM, or genomic DNA from mutant clones; PCR products were purified from agarose gels and sequenced to verify that the intron had indeed integrated into the correct target site (Supplementary Data S1).

### Southern blotting

Southern hybridisations used an intron-specific probe for the *ermB* marker to confirm integration of the group II intron into the desired target gene of mutants. The Digoxigenin (DIG) High Prime DNA Labelling and Detection Starter Kit II (Roche Diagnostics, Hertfordshire, UK) was used as per manufacturer instructions. A 900 bp probe for the *ermB* marker was created using ErmRAM-F and -R primers, and genomic DNA template from the *dnaK* mutant strain; 1 µg of this was labelled with DIG High Prime and a 20 µl aliquot hybridised to *Hin*dIII-digested DNA (500 ng) of *C. difficile* strains on a nylon membrane at 42 °C overnight. Anti-DIG antibody conjugated to alkaline phosphatase in combination with chemiluminescent substrate for alkaline phosphatase (CSPD) was used to develop the blot with exposure on a sheet of an X-ray film (Amersham Hyperfilm™ ECL, GE Healthcare, UK) for 5–10 min at room temperature, and subsequent development in a Kodak X-OMAT 1000 automated processor.

### Motility assays

Motility assays were performed as previously described by Tasteyre *et al*.^37^ Briefly, fresh BHIS broth with 0.175% technical agar No III (Oxoid) was placed in sterile glass test-tubes which were then stab inoculated with a clean toothpick using an actively growing colony of the respective strain. The tubes were incubated under strict anaerobic conditions at 37 °C for 48 h, following which growth of the mutant strain was visually compared to that of the wild-type. Motility assays were performed in three independent biological replicates.

### Electron microscopy

All microscopy-related work was performed at Ulster’s FEI Centre for Advanced Bioimaging. For SEM, glass cover slips (10 mm diameter) were cleaned using 10% Decon 90 detergent, rinsed and then soaked in a solution of 2% (v/v) 3-aminopropyltriethoxysilane (APES, Sigma Aldrich) and 100% methanol for 5 s. Following rinsing with 100% methanol and then deionised water, the cover slips were dried overnight at 37 °C. *C. difficile* cells from overnight cultures were collected by centrifugation (8000 × g) and washed with ice-cold PBS, and a drop of cell suspension was applied to the APES-coated coverslip and incubated at room temperature for 2 h. The liquid was removed and a drop of paraformaldehyde fixative (4% v/v in PBS) was added for 2 h. The fixative was removed and the cover slips were gently rinsed with PBS, followed by critical point drying (Polaron E5000 critical point drier) using acetone as an intermediary fluid. The cover slips were then attached to adhesive carbon pads on aluminium SEM stubs and sputter coated with an Au/Pd target in a Polaron E5100 sputter coater. Cells were visualised in an FEI (FEI, Eindhoven, Netherlands) Quanta™ ESEM under high vacuum at 30 kV using spot size 2 in secondary electron mode using an Everhart–Thornley detector. Images were acquired using the integrated imaging software.

For TEM, a clean glass microscope slide was dipped into a 0.5% (w/v in chloroform) formvar solution for 30 s. Following drying, the film was cut at the edges of the glass and floated on deionised water. A 200-mesh thin-bar copper grid (Agar scientific Ltd., UK) was placed on the floating formvar using forceps, and the film/grid was removed from the water using a Whatman No. 1 paper disc that was then completely dried at room temperature prior to use. Overnight cultures of *C. difficile* were harvested by centrifugation (3000 × g) and washed with ice-cold PBS. Formvar-coated grids were floated on a 50 µl drop of PBS-washed *C. difficile* cell suspension for 2 min, following which the excess cell suspension was wicked off the grids. The cells were then stained by floating the grid on a drop of 0.25% (w/v) phosphotungstic acid for 1 min prior to visualisation in an FEI Tecnai 12 transmission electron microscope (FEI, Eindhoven, Netherlands) using a lanthanum hexaboride (LaB_6_) source. The instrument was operated at 120 kV using spot size 1 and images acquired with a Megaview III camera and analySIS® image capture software (Soft Imaging Systems GmbH, Műnster, Germany).

### RNA isolation and qRT-PCR

RNA was extracted from late-log phase cultures, quality checked by agarose gel electrophoresis and ‘minus RT’-PCR with *tpi* primers, reverse transcribed using gene-specific primers (Table 3) and gene expression analysis was then performed as previously described^26–28^. The Relative Quantification (RelQuant) software (Roche Diagnostics) was used to assess gene expression levels, as per manufacturer instructions, and target gene expression was reported relative to the expression of the triose phosphate isomerase gene *tpi* (CD3172) in *C. difficile* 630 Δ*erm*.

**Table 3:** PCR primers.

Gene	Locus	Description	PPrimer	Sequence (5′ → 3′)	Binding position	Product size (bp)	Annealing temperature (°C)	Reference
*tpi*	CD3172	triosephosphate isomerase	tpi-Ftpi-R	GCAGGAAACTGGAAAATGCATAACAGATTGGCTCATATGCAACAAC	18–505	488	55	Lemèe *et al*.^74^
*groES*	CD0193	10 kDa chaperone	groES-FgroES-R	TACCAGGAGCAGCTAAAGAGTATCTCCCACTGTCAATTCC	80–187	108	55	This study
*groEL*	CD0194	60 kDa chaperonin	groEL-FgroEL-R	ATACTGAATTAGATGCTGTTGAAGTCTGAAGTAGTTTGCTCTACTTG	544–1069	526	53	Lemèe *et al*.^75^
*fliC*	CD0239	flagellin subunit	fliC-FfliC-R	GGGGTTAGAATCAAGAGAGCGTTTTAGCTGCATCTGTTCC	97–605	509	52	This study
*dnaJ*	CD2460	Chaperone protein	dnaJ-FdnaJ-R	TGGAAGAGCAAGAAGAAGAGTCAATCACTTCTCCAGTTCC	339–623	285	54	This study
*dnaK*	CD2461	chaperone protein	dnaK-FdnaK-R	TGTTAGAAGGTGGAGAAGCAGTTGCTTGTCTTTGAGCATC	53–389	337	56	This study
*grpE*	CD2462	heat shock protein	grpE-FgrpE-R	AAGCCGAATATGCAAACTACGATTTGCTTCAACTCCATCT	242–541	300	54	This study
*hrcA*	CD2463	heat-inducible transcription repressor	hrcA-FhrcA-R	ATGAGTAAAAGCGAATTGGATTGTCATTCATAGCAACCAA	238–440	202	55	This study

### Biofilm Assays

Biofilm formation assays were performed using a modified version of the method of O’Toole and Kolter^44^. Working anaerobically, overnight cultures of *C. difficile* 630Δ*erm* and of the *dnaK* mutant strain were diluted 100–f old into sterile BHIS broth, as well as into BHIS broths supplemented with 1%, 2% and 5% of 1 M filter-sterilised glucose solution (final concentrations of 0.38%, 0.56%, and 0.9%, respectively). Subsequently, 100 µl aliquots of each diluted culture was added to wells of a 96-well flat-bottom polystyrene microtitre plate (Orange Scientific, Alpha Technologies Ltd., UK) in 6 replicates. Replicates of uninoculated BHIS broth were included as the negative controls and to prevent evaporation, microtitre plates were incubated anaerobically at 37 °C with lids in place in a humidified plastic lunchbox–type container. Biofilm biomass was quantified at 24 h intervals for up to 3 days. Outside the cabinet, planktonic cells were washed from the wells using sterile deionised water and the plates air-dried for 30 min at room temperature. A 125 µl aliquot of 0.1% crystal violet solution (filter sterilised, in deionised water) was added to each well and allowed to stain the attached bacterial biomass for 15 min. The excess crystal violet was removed and the wells washed twice with deionised water, following which the plates were allowed to air dry at room temperature overnight. The next day, 200 µl of 95% ethanol was added to each well and the dye allowed to solubilise for 15 min. The crystal violet/ethanol solution in each well was mixed briefly using gentle pipetting and a 125 µl aliquot transferred to a separate 96-well microtitre plate to allow measurement of absorbance at 570 nm (*A*_570_) using a FLUOstar Omega microplate reader (BMG LabTech, UK). Background due to nonspecific staining by crystal violet was accounted for by subtracting the average values obtained from the wells containing the uninoculated negative controls.

### Equipment and settings

PCR gels were imaged under UV light in an AlphaImager™ 2200 (Alpha Innotech, CA, US) equipped with a 1.4 megapixel camera with 12 bit A/D, using default AlphaView Image Analysis Software settings, and exported in jpg format. The jpg images were imported into the GNU Image Manipulation Program (GiMP) 2.8 for generation of Fig. 1. Growth curve graphs shown in Fig. 2 were produced using MS Excel, individually exported in PDF and imported into GiMP 2.8 for construction and final labelling. Motility agar tubes were photographed using a Nikon D3 camera, 60 mm Nikkor macro lens, shutter speed 1/200 sec, aperture f/6.3, ISO 640, lit with studio flash and the resultant jpg images imported into GiMP 2.8 for construction and labelling of Fig. 3. Electron microscope images (tiff format) were imported into GiMP 2.8 for construction and labelling of Fig. 4. Gene expression data was used to construct a bar chart in MS Excel, prior to chart export in PDF; this was imported into GiMP 2.8 for final labelling of Fig. 5. Biofilm assay data was used to construct bar charts in MS Excel. The charts were individually exported in PDF and imported into GiMP for construction of Fig. 6. No alterations to brightness or contrast were made to any of the images during figure construction.

### Data availability

All data generated or analysed during this study are included in this published article (and in Supplementary Information files).

## Electronic supplementary material

Supplementary data

## References

[1] Rupnik M, Wilcox MH, Gerding DN (2009). *Clostridium difficile* infection: new developments in epidemiology and pathogenesis. Nat. Rev. Microbiol..

[2] Johanesen PA (2015). Disruption of the gut microbiome: *Clostridium difficile* infection and the threat of antibiotic resistance. Genes.

[3] Carter GP (2015). Defining the roles of TcdA and TcdB in localized gastrointestinal disease, systemic organ damage, and the host response during *Clostridium difficile* infections. mBio..

[4] Bartlett JG (2008). Historical perspectives on studies of *Clostridium difficile* and *C. difficile* infection. Clin. Infect. Dis..

[5] Freeman J (2015). Pan-European longitudinal surveillance of antibiotic resistance among prevalent *Clostridium difficile* ribotypes’ study group. Pan-European longitudinal surveillance of antibiotic resistance among prevalent *Clostridium difficile* ribotypes. Clin. Microbiol. Infect..

[6] Kociolek LK, Gerding DN (2016). Breakthroughs in the treatment and prevention of Clostridium difficileinfection. Nat. Rev. Gastroenterol. Hepatol..

[7] Spigaglia P (2016). Recent advances in the understanding of antibiotic resistance in *Clostridium difficile* infection. Ther. Adv. Infect. Dis..

[8] Saujet L, Monot M, Dupuy B, Soutourina O, Martin-Verstraete I (2011). The key sigma factor of transition phase, *SigH*, controls sporulation, metabolism, and virulence factor expression in *Clostridium difficile*. J. Bacteriol..

[9] Hutton ML, Mackin KE, Chakravorty A, Lyras D (2014). Small animal models for the study of *Clostridium difficile* disease pathogenesis. FEMS Microbiol. Lett..

[10] Bouillaut L, Dubois T, Sonenshein AL, Dupuy B (2015). Integration of metabolism and virulence in *Clostridium difficile*. Res. Microbiol..

[11] Sun X, Hirota SA (2015). The roles of host and pathogen factors and the innate immune response in the pathogenesis of *Clostridium difficile* infection. Mol. Immunol..

[12] Stabler RA (2006). Comparative phylogenomics of *Clostridium difficile* reveals clade specificity and microevolution of hypervirulent strains. J. Bacteriol..

[13] Stabler RA (2009). Comparative genome and phenotypic analysis of *Clostridium difficile* 027 strains provides insight into the evolution of a hypervirulent bacterium. Genome Biol..

[14] Stabler RA (2012). Macro and micro diversity of *Clostridium difficile* isolates from diverse sources and geographical locations. PLOS ONE.

[15] He M (2010). Evolutionary dynamics of *Clostridium difficile* over short and long time scales. Proc. Natl. Acad. Sci. USA.

[16] He M (2013). Emergence and global spread of epidemic healthcare-associated *Clostridium difficile*. Nat. Genet..

[17] Cartman, S. T., Heap, J. T., Kuehne, S. A., Cockayne, A. & Minton, N. P. The emergence of ‘hypervirulence’ In *Clostridium difficile*. *Int. J. Med. Microbiol*. **300**, 387–395 (2010). 10.1016/j.ijmm.2010.04.008 20547099

[18] Crowther GS (2015). Recurrence of dual-strain *Clostridium difficile* infection in an *in vitro* human gut model. J. Antimicrob. Chemother..

[19] Sebaihia M (2006). The multidrug-resistant human pathogen *Clostridium difficile* has a highly mobile, mosaic genome. Nat. Genet..

[20] Forgetta V (2011). Fourteen-genome comparison identifies DNA markers for severe-disease-associated strains of *Clostridium difficile*. J. Clin. Microbiol..

[21] Emerson JE, Stabler RA, Wren BW, Fairweather NF (2008). Microarray analysis of the transcriptional responses of *Clostridium difficile* to environmental and antibiotic stress. J. Med. Microbiol..

[22] Scaria J (2011). *Clostridium difficile* transcriptome analysis using pig ligated loop model reveals modulation of pathways not modulated *in vitro*. J. Infect. Dis..

[23] Scaria J (2013). Differential stress transcriptome landscape of historic and recently emerged hypervirulent strains of *Clostridium difficile* strains determined using RNA-seq. PLOS ONE..

[24] Chen JW (2013). Proteomic comparison of historic and recently emerged hypervirulent *Clostridium difficile* strains. J. Proteome. Res..

[25] Janoir C (2013). Adaptive strategies and pathogenesis of *Clostridium difficile* from *in vivo* transcriptomics. Infect. Immun..

[26] Jain S, Graham C, Graham RL, McMullan G, Ternan NG (2011). Quantitative proteomic analysis of the heat stress response in *Clostridium difficile* strain 630. J. Proteome. Res..

[27] Ternan NG, Jain S, Srivastava M, McMullan G (2012). Comparative transcriptional analysis of clinically relevant heat stress response in *Clostridium difficile* strain 630. PLOS ONE..

[28] Ternan NG, Jain S, Graham RLJ, McMullan G (2014). Semiquantitative analysis of clinical heat stress in *Clostridium difficile* strain 630 using a GeLC/MS workflow with emPAI quantitation. PLOS ONE..

[29] Xue C, Zhao X-Q, Liu C-G, Chen L-J, Bai F-W (2013). Prospective and development of butanol as an advanced biofuel. Biotechnol.Adv..

[30] Xue C, Zhao J, Chen L, Yang S-T, Bai F (2017). Recent advances and state-of-the-art strategies in strain and process engineering for biobutanol production by *Clostridium acetobutylicum*. Biotechnol. Adv..

[31] Li Q (2016). CRISPR-based genome editing and expression control systems in *Clostridium acetobutylicum* and *Clostridium beijerinckii*. Biotechnol. J..

[32] Heap JT, Pennington OJ, Cartman ST, Carter GP, Minton NP (2007). The ClosTron: A universal gene knock-out system for the genus. Clostridium. J. Microbiol. Meth..

[33] Heap JT (2010). The ClosTron: Mutagenesis in Clostridium refined and streamlined. J. Microbiol. Meth..

[34] Kuehne SA, Minton NP (2012). ClosTron-mediated engineering of Clostridium. Bioengineered..

[35] Underwood S (2009). Characterization of the sporulation initiation pathway of *Clostridium difficile* and its role in toxin production. J. Bacteriol..

[36] Susin MF, Baldini RL, Gueiros-Filho F, Gomes SL (2006). GroES/GroEL and DnaK/DnaJ have distinct roles in stress responses and during cell cycle progression in *Caulobacter crescentus*. J. Bacteriol..

[37] Tasteyre A, Barc MC, Collignon A, Boureau H, Karjalainen T (2001). Role of FliC and FliD flagellar proteins of *Clostridium difficile* in adherence and gut colonization. Infect. Immun..

[38] Tasteyre A (2000). A *Clostridium difficile* gene encoding flagellin. Microbiology.

[39] Twine SM (2000). Motility and flagellar glycosylation in *Clostridium difficile*. J. Bacteriol..

[40] Straus D, Walter W, Gross CA (1990). DnaK, DnaJ, and GrpE heat shock proteins negatively regulate heat shock gene expression by controlling the synthesis and stability of sigma 32. Genes Dev..

[41] Koch B, Kilstrup. M, Vogensen FK, Hammer K (1998). Induced levels of heat shock proteins in a *dnaK* mutant of *Lactococcus lactis*. J. Bacteriol..

[42] Zhao K, Liu M, Burgess RR (2005). The global transcriptional response of *Escherichia coli* to induced sigma 32 protein involves sigma 32 regulon activation followed by inactivation and degradation of sigma 32 *in vivo*. J. Biol. Chem..

[43] Deligianni E (2010). *Pseudomonas aeruginosa* cystic fibrosis isolates of similar RAPD genotype exhibit diversity in biofilm forming ability *in vitro*. BMC Microbiol..

[44] O’Toole GA, Kolter R (1998). Initiation of biofilm formation in *Pseudomonas fluorescens* WCS365 proceeds via multiple, convergent signalling pathways: a genetic analysis. Mol. Microbiol..

[45] Varga JJ, Therit B, Melville SB (2008). Type IV pili and the CcpA protein are needed for maximal biofilm formation by the Gram-positive anaerobic pathogen *Clostridium perfringens*. Infect. Immun..

[46] Selby K (2011). Important role of class I heat shock genes *hrcA* and *dnaK* in the heat shock response and the response to pH and NaCl stress of group I *Clostridium botulinum* strain ATCC 3502. Appl. Environ. Microbiol..

[47] Itikawa H, Ryu J (1979). Isolation and characterization of a temperature-sensitive *dnaK* mutant of *Escherichia coli* B. J. Bacteriol..

[48] Paek KH, Walker GC (1987). *Escherichia coli dnaK* null mutants are inviable at high temperature. J. Bacteriol..

[49] Bukau B, Walker GC (1989). Cellular defects caused by deletion of the *Escherichia coli dnaK* gene indicate roles for heat shock protein in normal metabolism. J. Bacteriol..

[50] Singh VK (2007). Role for *dnaK* locus in tolerance of multiple stresses in *Staphylococcus aureus*. Microbiology.

[51] Schulz A, Tzschaschel B, Schumann W (1995). Isolation and analysis of mutants of the *dnaK* operon of *Bacillus subtilis*. Mol. Microbiol..

[52] McCarty JS, Walker GC (1994). DnaK mutants defective in ATPase activity are defective in negative regulation of the heat shock response: expression of mutant DnaK proteins results in filamentation. J. Bacteriol..

[53] Margolin W (2005). FtsZ and the division of prokaryotic cells and organelles. Nat. Rev. Mol. Cell Biol..

[54] Sugimoto S, Saruwatari K, Higashi C, Sonomoto K (1990). The proper ratio of GrpE to DnaK is important for protein quality control by the DnaK-DnaJ-GrpE chaperone system and for cell division. Microbiology.

[55] Homuth G, Masuda S, Mogk A, Kobayashi Y, Schumann W (1997). The *dnaK* operon of *Bacillus subtilis* is heptacistronic. J. Bacteriol..

[56] Homuth G, Mogk A, Schumann W (1999). Post-transcriptional regulation of the *Bacillus subtilis dnaK* operon. Mol. Microbiol..

[57] Takaya A, Tomoyasu T, Matsui H, Yamamoto T (2004). The DnaK/DnaJ chaperone machinery of *Salmonella enterica* serovar Typhimurium is essential for invasion of epithelial cells and survival within macrophages, leading to systemic infection. Inf. Immunity..

[58] Hecker M, Pané-Farré J, Völker U (2007). SigB-dependent general stress response in *Bacillus subtilis* and related gram-positive bacteria. Annu. Rev. Microbiol..

[59] Narberhaus F (1999). Negative regulation of bacterial heat shock genes. Mol. Microbiol..

[60] Helmann JD (2001). Global transcriptional response of *Bacillus subtilis* to heat shock. J. Bacteriol..

[61] Mogk A (1997). The GroE chaperonin machine is a major modulator of the CIRCE heat shock regulon of *Bacillus subtilis*. EMBO J..

[62] Schulz A, Schumann W (1996). *hrcA*, the first gene of the *Bacillus subtilis dnaK* operon encodes a negative regulator of class I heat shock genes. J. Bacteriol..

[63] Poutanen SM, Simor. AE (2004). *Clostridium difficile*-associated diarrhea in adults. CMAJ.

[64] Shi W, Zhou Y, Wild J, Adler J, Gross CA (1992). DnaK, DnaJ, and GrpE are required for flagellum synthesis in. Escherichia coli. J. Bacteriol..

[65] Hossain MM, Tsuyumu S (2006). Flagella-mediated motility is required for biofilm formation by *Erwinia carotovora* subsp. *carotovora*. J. Gen. Plant Pathol..

[66] Houry A, Briandet R, Aymerich S, Gohar M (2010). Involvement of motility and flagella in *Bacillus cereus* biofilm formation. Microbiology.

[67] Hennequin CF (2001). GroEL (Hsp60) of *Clostridium difficile* is involved in cell adherence. Microbiology.

[68] Ensgraber M, Loos M (1992). A 66-kilodalton heat shock protein of *Salmonella typhimurium* is responsible for binding of the bacterium to intestinal mucus. Infect. Immun..

[69] Phadnis SH (1996). Surface localization of *Helicobacter pylori* urease and a heat shock protein homolog requires bacterial autolysis. Infect. Immun..

[70] Bergonzelli G (2006). GroEL of *Lactobacillus johnsonii* La1 (NCC 533) is cell surface associated: potential role in interactions with the host and the gastric pathogen *Helicobacter pylori*. Infect. Immun..

[71] Jain S, Graham RL, McMullan G, Ternan NG (2010). Proteomic analysis of the insoluble subproteome of *Clostridium difficile* strain 630. FEMS Microbiol. Lett..

[72] Hussain HA, Roberts AP, Mullany P (2005). Generation of an erythromycin-sensitive derivative of *Clostridium difficile* strain 630 (630Deltaerm) and demonstration that the conjugative transposon Tn916DeltaE enters the genome of this strain at multiple sites. J. Med. Microbiol..

[73] Heap JT, Pennington OJ, Cartman ST, Minton NP (2009). A modular system for *Clostridium* shuttle plasmids. J. Microbiol. Meth..

[74] Lemée L, Dhalluin A, Pestel-Caron M, Lemeland J, Pons J (2004). Multilocus sequence typing analysis of human and animal *Clostridium difficile* isolates of various toxigenic types. J. Clin. Microbiol..

[75] Lemée L (2005). Multilocus sequence analysis and comparative evolution of virulence-associated genes and housekeeping genes of *Clostridium difficile*. Microbiol..

