# Supplementary material for: Inactivation of the dnaK gene in Clostridium difficile 630 Δerm yields a temperature-sensitive phenotype and increases biofilm-forming ability
Source: Sci Rep. 2017 Dec 13;7:17522. doi: 10.1038/s41598-017-17583-9 (PMC5727486; doi:10.1038/s41598-017-17583-9)
Supplement: Supplementary file 1 — Supplementary data [file 41598_2017_17583_MOESM1_ESM.pdf]

Inactivation of the *dnaK* gene in *Clostridium difficile* 630 Δ*erm* yields a temperature-sensitive phenotype and increases biofilm-forming ability

Shailesh Jain, Deborah Smyth, Barry M. G. O'Hagan, John T. Heap[1], Geoff McMullan[2], Nigel P. Minton[3] and Nigel G. Ternan*

Nutrition **I**nnovation **C**entre for **F**ood and **HE**alth (NICHE), School of Biomedical Sciences, University of Ulster, Coleraine, Co. Londonderry, N. Ireland, BT52 1SA, U. K.

[1]Centre for Synthetic Biology and Innovation, Department of Life Sciences, Imperial College London, South Kensington Campus, London SW7 2AZ, U. K.

[2]Institute for Global Food Security, School of Biological Sciences, Queen's University Belfast, N. Ireland, United Kingdom BT9 7BL, U. K.

[3]Clostridia Research Group, BBSRC/EPSRC Synthetic Biology Research Centre (SBRC), University of Nottingham, University Park, Nottingham NG7 2RD, U. K.

Supplementary data S1

*Hin*dIII

AAAAAAGCTTATAATTATCCTTAAATTCCTTCTTAGTGCGCCCAGATAGGGTGTTAAGTCAAGTAGTTTAAGG
TACTACTCTGTAAGATAACACAGAAAACAGCCAACCTAACCGAAAAGCGAAAGCTGATACGGGAACAGAGCA
CGGTTGGAAAGCGATGAGTTACCTAAAGACAATCGGGTACGACTGAGTCGCAATGTTAATCAGATATAAGGT
ATAAGTTGTGTTTACTGAACGCAAGTTTCTAATTTCGATTGAATTTCGATAGAGGAAAGTGTCTGAAACCTCTA
GTACAAAGAAAGGTAAGTTAGCTAAGAAGACTTATCTGTTATCACCACATTTGTACAATCTG

*Bsr*GI

The 353 bp targeting region for *dnaK* gene from pMTL007C-E2. The unique *Hin*dIII and *Bsr*GI restriction sites are shown in red (purple line indicates cut site), IBS primer sequence is highlighted in yellow, EBS2 primer sequence is highlighted in green, and the reverse complement of EBS1d primer sequence is highlighted in blue.

PCR1 across intron/exon junction. EBS universal and Gene specific R1 (for antisense insertion) yields 428bp product from the dnaK mutant only as per Fig. 1a, lane 2.

- Sequence derived from sequencing using EBS universal primer:

>PCF-R1SJ-EBS-(PCR_1_EBS.ab1) 227nt
ATTGCGACTCAGTCGTACCCGATTGTCTTTAGGTAACTCATCGCTTTCCAACCGTGCTCTGTTCCCGTATCAGCT
TTCGCTTTTCGGTTAGGTTGGCTGTTTTCTGTGTTATCTTACAGAGTAGTACCTTAAACTACTTGACTTAACACC
CTATCTGGGCGCACTAAGAAAGAATTATCATCAACAATGAGTTCAAACATAAACTTACCTTTCATACCTGCTAC
AGA

When this sequence analysed using BlastN using the *C. difficile* 630 genome, nucleotides 164-226 match AM180355.1 (2,840,918..2,840,980) – this is dnaK, 62nucleotides of seq (underlined).

When analysed by BlastN in the nr db, nucleotides 1-165 match pMTL007-CE2, 165 nucleotides of sequence. Highlighted nucleotide is position 723.

- Sequence derived from sequencing using Cdi-dnaK-R primer:

>PCF-R1SJ-SR (PCR_1_SR.ab1) 251nt

ACTCTTGTTGATGATAATTCTTTCTTAGTGGCGCCCAGATNGGGTGGTTAAGTCAAGTAGTTTAAGGTACTCCT
CTGTAAGATAACACAGAAAACAGCCAACCTAACCGAATAGCGAAAGCTGATACGGGAACAGAGCACGGTCG
GAAAGCGATGAGTTACCTAAAGACAATCGGGTACGACCGAGTCGCAACGTTAATCAGATATAAGGTATAAGT
TGTGTTTACTGAACGCAAGCTCTTAATTTCGGAA

Reverse complement:

>R1-rev-c

TTCCGAAATTAAGAGCTTGCGTTCAGTAAACACAACTTATACCTTATATCTGATTAACGTTGCGACTCGGTCGT
ACCCGATTGTCTTTAGGTAACTCATCGCTTTCCGACCGTGCTCTGTTCCCGTATCAGCTTTCGCTATTCGGTTAG

Supplementary data S1

GTTGGCTGTTTTCTGTGTTATCTTACAGAGGAGTACCTTAAACTACTTGACTTAACCACCCNATCTGGGCGCCA
CTAAGAAAGAATTATCATCAACAAGAGT

Upon analysis as above, nucleotides 3-225 match pMTL007-CE2 (221nt), and nucleotides 226-251 matches dnaK (25nt, underlined).

Supplementary data S1

PCR across the other intron-exon junction using ErmRAM-R and Cdi-dnaK-F primers generated a 1300 bp product from the dnaK mutant only as per Fig 1c (iii) lane 2.

- Sequence derived from sequencing using Cdi-dnaK-F primer:

PCR_3_SF.ab1 (1016nt)
AATAGTATAGATTATTTAGCAGAGGAATTTAAAAAAGCTGAAGGTGTAGATTTAAGAAATGATAAAATGGCTC
TTCAAAGATTAAAAGAAGCAGCAGAAAAGGCGTGAAGTAGGGAGGTACCGCCTTGTTCACATTACTGTGACT
GGTTTGCACCACCCTCTTCGGGAACCGTACGTACCCCTCTCGGAGTATACGGCTCTGTTATTGTTCGTTCGTAA
AAATTCACTGTCGACATTCACTTGTGTTTATGAATCACGTGACGATGACAATGAAAGCATACAACAAGAGTTTT
ACGTTGTTTCGCTATCATTGCCATTTCCCAACGCGTGAAGTTCCTATTCTCTAGAAAGTATAGGAACTTCTATAT
TGATAAAAATAATAATAGTGGGTATAATTAAGTTGTTAGAGAAAACGTATAAATTAGGAGGGATTCATATGG
ACCCAAGAGATGCTGGTGCTTCTGGTGCTGGTATGAACAAAAATATAAAATATTCTCAAAACTTTTTAACGAGT
GAAAAAGTACTCAACCAAATAATAAAACAATTGAATTTAAAAGAAACCGATACCGTTTACGAAATTGGAACAG
GTAAAGGGCATTTAACGACGAAACTGGCTAAAATAAGTAAACAGGTAACGTCTATTGAATTAGACAGTCATCT
ATTCAACTTATCGTCAGAAAAATTAAAACTGAATACTCGTGTCACTTTAATTCACCAAGATATTCTACAGTTTCA
ATTCCCTAACAAACAGAGGTATAAAATTGTTGGGAGTATTCCTTACCATTTAAGCACACAAATTATTAAAAAAG
TGGTTTTTGAAAGCCATGCGTCTGACATCTATCTGATTGTTGAAGAAGGATTCTACAAGCGTACCTTGNATATT
CACCGAACACTNGGGTTGCTCTTGCACACTCAAGTCTCGATTCAGCAATTGCTTAAGCTGCCAGCGGAATGCT
TTCATCCTAAACCAAAAGTAAACAGTGTCNTAATAAAACTTACCCGCCATACCACAGATG

When this sequence was analysed using BlastN and the C. difficile 630 genome, nucleotides 1-104 match AM180355.1 – this is dnaK, 104nucleotides of seq (underlined).

When analysed by BlastN in the nr db, nucleotides 105-1016 match pMTL007-CE2, 912 nucleotides of sequence.

- Sequence derived from sequencing using ErmRAM-R primer:

PCR_3_RAM_R.ab1 REV COMP (1012nt) – matched pMTL007-CE2
TTCGGGAACCGTACGTACCCCTCTCGGAGTATACGGCTCTGTTATNNTTCGTTCGTAAAAATTCACTGTCGACA
TTCACTTGTGTTTATGAATCACGTGACGATGACAATGAAAGCATACAACAAGAGTTTTACGTTGTTTCGCTATC
ATTGCCATTTCCCAACGCGTGAAGTTCCTATTCTCTAGAAAGTATAGGAACTTCTATATTGATAAAAATAATAA
TAGTGGGTATAATTAAGTTGTTAGAGAAAACGTATAAATTAGGAGGGATTCATATGGACCCAAGAGATGCTG
GTGCTTCTGGTGCTGGTATGAACAAAAATATAAAATATTCTCAAAACTTTTTAACGAGTGAAAAAGTACTCAAC
CAAATAATAAAACAATTGAATTTAAAAGAAACCGATACCGTTTACGAAATTGGAACAGGTAAAGGGCATTTAA
CGACGAAACTGGCTAAAATAAGTAAACAGGTAACGTCTATTGAATTAGACAGTCATCTATTCAACTTATCGTCA
GAAAAATTAAAACTGAATACTCGTGTCACTTTAATTCACCAAGATATTCTACAGTTTCAATTCCCTAACAAACAG
AGGTATAAAATTGTTGGGAGTATTCCTTACCATTTAAGCACACAAATTATTAAAAAAGTGGTTTTTGAAAGCCA
TGCGTCTGACATCTATCTGATTGTTGAAGAAGGATTCTACAAGCGTACCTTGGATATTCACCGAACACTAGGGT
TGCTCTTGCACACTCAAGTCTCGATTCAGCAATTGCTTAAGCTGCCAGCGGAATGCTTTCATCCTAAACCAAAA
GTAAACAGTGTCTTAATAAAACTTACCCGCCATACCACAGATGTTCCAGATAAATATTGGAAGCTATATACGTA
CTTTGTTTCAAAATGGGTCAATCGAGAATATCGTCAACTGTTTACTAAAAATCAGTTTCATCAAGCAATGAAAC
ACGCCAAAGTAAACAATTTAAGTACCGTTACTTATGAGCAAGTATTGTCTAT

When analysed by BlastN in the nr db, nucleotides 1-1012 match pMTL007-CE2, 912 nucleotides of sequence. Red highlighted nucleotides indicate overlap between sequences.

Supplementary data S2

Figure 2 growth curve raw attenuance data

Derm 37°C<br>Time (h)	Biol rep 1<br>D650nm	Biol rep 2<br>D650nm	Biol rep 3<br>D650nm	Average<br>D650nm	SEM
0	0.051	0.053	0.054	0.0526667	0.0008819
1	0.048	0.045	0.055	0.0493333	0.0029627
2	0.11	0.098	0.1	0.1026667	0.0037118
3	0.166	0.165	0.164	0.165	0.0005774
4	0.29	0.296	0.292	0.2926667	0.0017638
5	0.595	0.58	0.585	0.5866667	0.0044096
6	0.836	0.848	0.85	0.8446667	0.0043716
7	1.192	1.185	1.17	1.1823333	0.0064893
8	1.55	1.3	1.316	1.3886667	0.0807988
9	1.53	1.65	1.57	1.5833333	0.0352767
10	1.56	1.5	1.62	1.56	0.034641

Derm 30°C<br>Time (h)	Biol rep 1<br>D650nm	Biol rep 2<br>D650nm	Biol rep 3<br>D650nm	Average<br>D650nm	SEM
0	0.033	0.03	0.036	0.033	0.0017321
1	0.045	0.04	0.043	0.0426667	0.001453
2	0.076	0.076	0.075	0.0756667	0.0003333
3	0.13	0.15	0.145	0.1416667	0.0060093
4	0.245	0.25	0.246	0.247	0.0015275
5	0.408	0.42	0.412	0.4133333	0.0035277
6	0.604	0.612	0.632	0.616	0.0083267
7	0.816	0.82	0.792	0.8093333	0.0087433
8	0.976	0.936	0.932	0.948	0.0140475
9	1.092	1.092	1.044	1.076	0.016
10	1.1	0.96	0.98	1.0133333	0.0437163

Derm 41°C<br>Time (h)	Biol rep 1<br>D650nm	Biol rep 2<br>D650nm	Biol rep 3<br>D650nm	Average<br>D650nm	SEM
0	0.048	0.052	0.051	0.0503333	0.0012019
1	0.046	0.042	0.045	0.0443333	0.0012019
2	0.098	0.1	0.099	0.099	0.0005774
3	0.166	0.172	0.173	0.1703333	0.0021858
4	0.293	0.298	0.304	0.2983333	0.0031798
5	0.628	0.63	0.62	0.626	0.0030551
6	0.916	0.964	0.892	0.924	0.021166
7	1.224	1.244	1.276	1.248	0.0151438
8	1.79	1.675	1.655	1.7066667	0.0420648
9	1.5	1.565	1.57	1.545	0.0225462
10	1.55	1.485	1.525	1.52	0.0189297

Derm 45°C<br>Time (h)	Biol rep 1<br>D650nm	Biol rep 2<br>D650nm	Biol rep 3<br>D650nm	Average<br>D650nm	SEM
0	0.025	0.036	0.028	0.0296667	0.003283
1	0.04	0.042	0.039	0.0403333	0.0008819
2	0.079	0.082	0.07	0.077	0.0036056
3	0.145	0.13	0.125	0.1333333	0.0060093
4	0.253	0.258	0.22	0.2436667	0.011921
5	0.508	0.516	0.44	0.488	0.0241109
6	0.728	0.656	0.688	0.6906667	0.0208273
7	0.772	0.764	0.752	0.7626667	0.0058119
8	0.8	0.832	0.784	0.8053333	0.0141107
9	0.7	0.724	0.744	0.7226667	0.0127192
10	0.65	0.68	0.66	0.6633333	0.0088192

Supplementary data S2

Figure 2 growth curve raw attenuance data

Mutant 37°C<br>Time (h)	Biol rep 1<br>D650nm	Biol rep 2<br>D650nm	Biol rep 3<br>D650nm	Average<br>D650nm	SEM
0	0.039	0.032	0.027	0.0326667	0.0034801
1	0.033	0.03	0.032	0.0316667	0.0008819
2	0.09	0.098	0.1	0.096	0.0030551
3	0.162	0.161	0.155	0.1593333	0.0021858
4	0.258	0.265	0.25	0.2576667	0.0043333
5	0.347	0.347	0.345	0.3463333	0.0006667
6	0.468	0.464	0.46	0.464	0.0023094
7	0.588	0.612	0.568	0.5893333	0.0127192
8	0.665	0.69	0.695	0.6833333	0.0092796
9	0.7	0.75	0.765	0.7383333	0.0196497
10	0.715	0.7	0.675	0.6966667	0.0116667

Mutant 30°C<br>Time (h)	Biol rep 1<br>D650nm	Biol rep 2<br>D650nm	Biol rep 3<br>D650nm	Average<br>D650nm	SEM
0	0.025	0.03	0.028	0.0276667	0.001453
1	0.047	0.054	0.054	0.0516667	0.0023333
2	0.068	0.07	0.076	0.0713333	0.0024037
3	0.12	0.11	0.115	0.115	0.0028868
4	0.2	0.211	0.205	0.2053333	0.0031798
5	0.296	0.324	0.292	0.304	0.0100664
6	0.4	0.412	0.416	0.4093333	0.0048074
7	0.488	0.532	0.496	0.5053333	0.0135319
8	0.664	0.624	0.6	0.6293333	0.0186667
9	0.724	0.696	0.74	0.72	0.0128582
10	0.69	0.72	0.66	0.69	0.0173205

Mutant 41°C<br>Time (h)	Biol rep 1<br>D650nm	Biol rep 2<br>D650nm	Biol rep 3<br>D650nm	Average<br>D650nm	SEM
0	0.038	0.032	0.03	0.0333333	0.0024037
1	0.037	0.036	0.03	0.0343333	0.0021858
2	0.09	0.095	0.092	0.0923333	0.001453
3	0.178	0.188	0.166	0.1773333	0.0063596
4	0.248	0.238	0.241	0.2423333	0.0029627
5	0.338	0.348	0.34	0.342	0.0030551
6	0.428	0.42	0.416	0.4213333	0.0035277
7	0.42	0.452	0.6	0.4906667	0.0554417
8	0.525	0.53	0.475	0.51	0.0175594
9	0.515	0.525	0.505	0.515	0.0057735
10	0.58	0.505	0.5	0.5283333	0.0258736

Mutant 45°C<br>Time (h)	Biol rep 1<br>D650nm	Biol rep 2<br>D650nm	Biol rep 3<br>D650nm	Average<br>D650nm	SEM
0	0.029	0.028	0.021	0.026	0.0025166
1	0.048	0.025	0.035	0.036	0.0066583
2	0.074	0.071	0.072	0.0723333	0.0008819
3	0.125	0.105	0.115	0.115	0.0057735
4	0.219	0.215	0.21	0.2146667	0.0026034
5	0.328	0.324	0.292	0.3146667	0.011392
6	0.312	0.292	0.32	0.308	0.0083267
7	0.288	0.28	0.28	0.2826667	0.0026667
8	0.216	0.256	0.244	0.2386667	0.0118509
9	0.124	0.184	0.176	0.1613333	0.018809
10	0.14	0.12	0.11	0.1233333	0.0088192

Supplementary data S3

qRT-PCR expression ratios - CD630Derm versus dnaK mutant

**GroESL**		Rep 1	Rep 2	Average	Std Dev
WT normalized ratio				1	
groEL		4.79	3.95	4.37	0.42
groES		4.004	2.756	3.38	0.624

**dnaK**		Rep 1	Rep 2	Average	Std Dev
WT normalized ratio				1	
hrcA		1.3145	1.056	1.18525	0.182787
grpE		4.256	3.421	3.8385	0.590434
dnaK	1.71	1.61	2.23	1.85	0.332866
dnaJ		0.296	0.212	0.254	0.059397

fliC		Rep 1	Rep 2	Average	Std Dev
WT normalized ratio				1	
fliC		0.346	0.1638	0.2549	0.128835

**Gene**	**ratio mut/wt**	**log 2 (ratio)**	**fold change (2 to power of log ratio)**	**direction**
tpi	1	0	1	N/A
fliC	0.2549	-1.971996722	3.923107101	down
groEL	4.37	2.12763328	4.37	up
groES	3.38	1.757023247	3.38	up
hrcA	1.18525	0.245191393	1.18525	up
grpE	3.8385	1.940542648	3.8385	up
dnaK	1.85	0.887525271	1.85	up
dnaJ	0.254	-1.977099598	3.937007874	down

gene	**fold change**	**St Dev**
fliC	-3.9231071	0.128834856
groEL	4.37	0.42
groES	3.38	0.624
hrcA	1.18525	0.182787
grpE	3.8385	0.590434
dnaK	1.85	0.332866
dnaJ	-3.9370079	0.059397

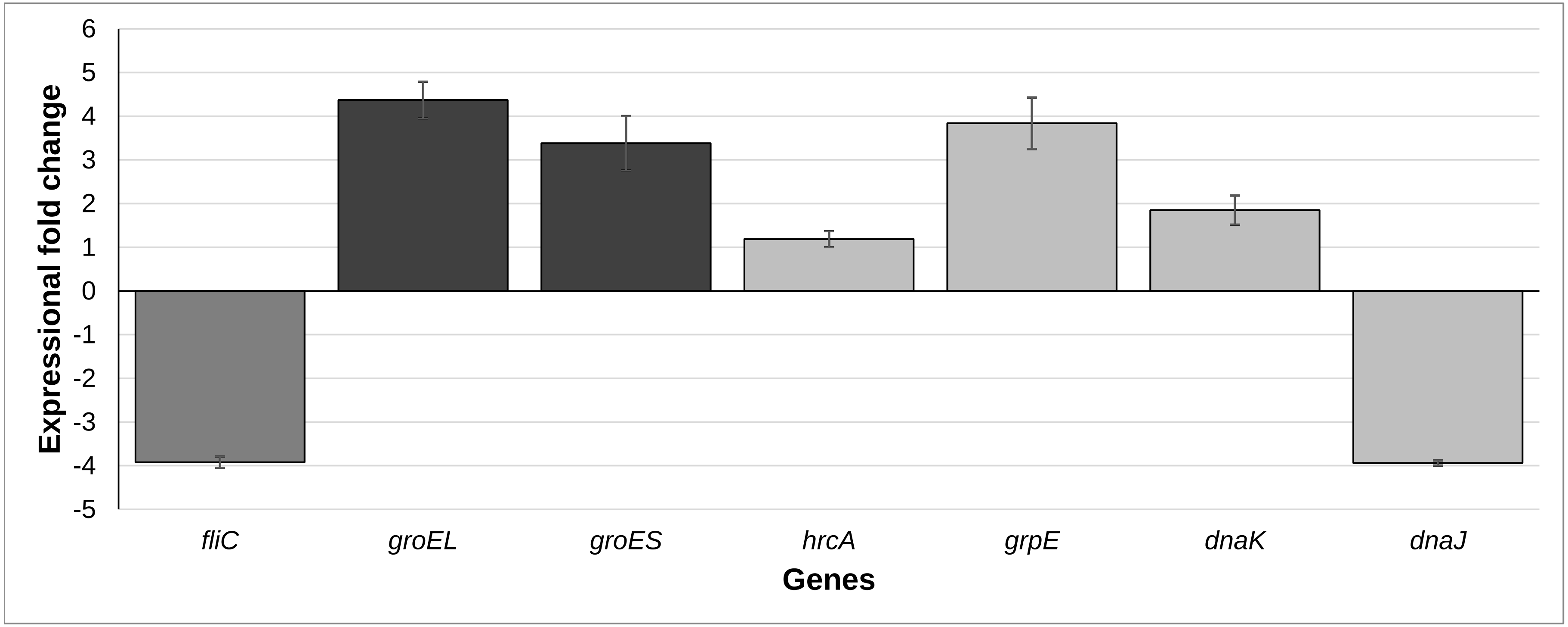

6
5
4
3
2
1
0
-1
-2
-3
-4
-5
Expressional fold change
fliC
groEL
groES
hrcA
grpE
dnaK
dnaJ
Genes

Supplementary Data S4

Raw A570 data, 24h

Biol Rep 1

		Derm				Mutant			
	Media blank	BHIs	0.38% Glu	0.56% Glu	0.9% Glu	BHIs	0.38% Glu	0.56% Glu	0.9% Glu
	0.0835	0.1339	0.1267	0.114	0.1345	0.2305	0.838	0.906	0.7187
	0.0938	0.1227	0.1712	0.1112	0.1721	0.1644	0.3278	0.6699	0.5974
	0.1054	0.1096	0.1116	0.1072	0.1735	0.2634	0.8942	0.9074	0.9666
	0.0938	0.106	0.1071	0.1077	0.15	0.2037	0.8791	0.9646	0.8475
	0.0929	0.1056	0.1047	0.1112	0.1747	0.2479	0.7211	0.6202	0.98
	0.0957	0.1289	0.1088	0.1414	0.1464	0.2251	0.7139	0.5822	0.5933
Mean of 6 tech reps	0.094183333	0.1177833	0.1216833	0.11545	0.1585333	0.2225	0.7290167	0.77505	0.7839167
corrected for media blank		0.0236003	0.0275003	0.021267	0.06435	0.128317	0.6348337	0.680867	0.689733

Biol Rep 2

		Derm				Mutant			
	Blank	BHIs	0.38% Glu	0.56% Glu	0.9% Glu	BHIs	0.38% Glu	0.56% Glu	0.9% Glu
	0.0835	0.12	0.1215	0.1156	0.1431	0.1765	0.8537	0.7623	0.6526
	0.0938	0.1245	0.0904	0.1469	0.1218	0.1733	0.6024	0.4973	0.6
	0.08	0.135	0.0929	0.1412	0.1197	0.2448	0.3558	0.5271	0.7944
	0.1	0.1289	0.0986	0.1081	0.1224	0.3212	0.4219	0.5514	0.9149
	0.111	0.131	0.1021	0.1199	0.1658	0.2877	0.4512	0.7416	0.8466
	0.0957	0.1317	0.1152	0.1426	0.1482	0.2799	0.5986	0.3988	0.4849
Mean of 6 tech reps	0.094	0.1285167	0.10345	0.12905	0.1368333	0.2472333	0.5472667	0.57975	0.7155667
corrected for media blank		0.0345167	0.00945	0.03505	0.0428333	0.1532333	0.4532667	0.48575	0.621567

Biol Rep 3

		Derm				Mutant			
	Blank	BHIs	0.38% Glu	0.56% Glu	0.9% Glu	BHIs	0.38% Glu	0.56% Glu	0.9% Glu
	0.108	0.1142	0.1091	0.1343	0.1197	0.2122	0.7318	0.29	0.7554
	0.1071	0.1086	0.1351	0.1307	0.1014	0.1125	0.6834	0.798	0.4423
	0.0905	0.0863	0.1201	0.1406	0.1296	0.3019	0.3081	0.4347	0.6209
	0.1107	0.1112	0.112	0.1444	0.228	0.2425	0.7591	0.7441	0.575
	0.0978	0.1163	0.101	0.1249	0.1539	0.2138	0.2858	0.2797	0.3206
	0.108	0.1383	0.1145	0.1479	0.142	0.1825	0.5737	0.4697	0.4165
Mean of 6 tech reps	0.103683333	0.1124833	0.1153	0.1371333	0.1457667	0.2109	0.5569833	0.5027	0.5217833
corrected for media blank		0.0088003	0.011617	0.0334503	0.0420837	0.107217	0.4533003	0.399017	0.4181003

Supplementary Data S4

Raw A570 data, 48h

	Blank	Derm BHIs	Derm 0.38% Glu	Derm 0.56% Glu	Derm 0.9% Glu	Mutant BHIs	Mutant 0.38% Glu	Mutant 0.56% Glu	Mutant 0.9% Glu
**Biol Rep 1**									
	0.0809	0.1094	0.0997	0.1043	0.2168	0.5998	1.1722	1.3669	1.604
	0.1083	0.1269	0.1236	0.1459	0.2426	0.5512	1.0107	1.4824	1.5
	0.0934	0.1083	0.1192	0.15	0.2666	0.4	1.2508	1.5931	1.1569
	0.1078	0.1385	0.1498	0.1471	0.1971	0.4896	0.9312	1.1749	1.9
	0.086	0.1878	0.1435	0.1682	0.2062	0.5	1.1483	1.7574	1.6092
	0.0947	0.1257	0.1115	0.1249	0.2116	0.4357	1.0887	1.2676	1.8
Mean of 6 tech reps	0.095183333	0.1327667	0.12455	0.1400667	0.2234833	0.49605	1.1003167	1.4403833	1.5950167
corrected for media blank		0.0375837	0.029367	0.0448837	0.1283003	0.400867	1.0051337	1.3452003	1.4998337
**Biol Rep 2**									
	0.0927	0.1338	0.1168	0.1294	0.1295	0.2406	0.9978	1.5974	1.7827
	0.0992	0.1071	0.1817	0.1241	0.1888	0.2453	0.8131	1.3465	1.4717
	0.098	0.1251	0.1767	0.1666	0.2152	0.2544	0.7342	1.4788	1.8219
	0.1259	0.1244	0.1126	0.127	0.1914	0.3215	0.8	0.9	1.3356
	0.0891	0.1511	0.1034	0.148	0.1826	0.266	0.9	0.7834	1.5395
	0.0893	0.1243	0.1068	0.1752	0.138	0.4256	0.7389	1.1168	1.7
Mean of 6 tech reps	0.099033333	0.1276333	0.133	0.14505	0.17425	0.2922333	0.8306667	1.2038167	1.6085667
corrected for media blank		0.0286003	0.033967	0.046017	0.075217	0.1932003	0.7316337	1.1047837	1.5095337
**Biol Rep 3**									
	0.0861	0.1213	0.1056	0.2215	0.1921	0.3954	1.3683	1.6718	1.0282
	0.0957	0.0945	0.1147	0.2643	0.1875	0.4374	1.3124	0.941	1.2775
	0.0955	0.1428	0.1086	0.1593	0.1928	0.1963	0.9027	0.823	1.0414
	0.1	0.1259	0.1041	0.2021	0.1364	0.4623	1.1815	1.3796	1.2796
	0.0845	0.1021	0.1382	0.129	0.1836	0.1557	1.4211	1.0891	1.1686
	0.0965	0.1278	0.1417	0.1443	0.1613	0.3532	1.2272	1	1.3
Mean of 6 tech reps	0.09305	0.1190667	0.1188167	0.18675	0.1756167	0.3333833	1.2355333	1.15075	1.18255
corrected for media blank		0.0260167	0.0257667	0.0937	0.0825667	0.2403333	1.1424833	1.0577	1.0895

Supplementary Data S4

Raw A570 data, 72h

Biol Rep 1

		Derm				Mutant			
	Blank	BHIs	0.38% Glu	0.56% Glu	0.9% Glu	BHIs	0.38% Glu	0.56% Glu	0.9% Glu
	0.086	0.1416	0.1276	0.2176	0.2308	0.6316	1.3661	1.4958	1.7797
	0.1235	0.1255	0.1328	0.2142	0.2962	0.7266	1.392	1.5448	1.4926
	0.1202	0.167	0.1409	0.2497	0.3107	0.5375	1.1144	1.4902	1.9047
	0.1364	0.2246	0.1857	0.2552	0.268	0.6992	1.5085	1.4296	1.9282
	0.0914	0.1196	0.1738	0.2767	0.3395	0.6986	1.2321	1.2859	1.4705
	0.106	0.17	0.3107	0.2069	0.1566	0.8109	1.0073	1.2043	1.3644
Mean of 6 tech reps	0.1105833	0.15805	0.1785833	0.2367167	0.2669667	0.6840667	1.2700667	1.4084333	1.6566833
corrected for media blank		0.047467	0.0680003	0.1261337	0.1563837	0.5734837	1.1594837	1.2978503	1.5461003

Biol Rep 2

		Derm				Mutant			
	Blank	BHIs	0.38% Glu	0.56% Glu	0.9% Glu	BHIs	0.38% Glu	0.56% Glu	0.9% Glu
	0.0901	0.1687	0.1764	0.1439	0.1344	0.8207	0.9521	1.2593	1.8681
	0.1153	0.1546	0.1749	0.1703	0.2251	1.0083	0.9336	1.5275	1.4838
	0.0855	0.1717	0.1771	0.1709	0.1415	0.9075	1.2782	1.4761	1.6154
	0.116	0.1611	0.2036	0.1754	0.1795	0.701	0.9955	1.3274	1.7936
	0.1073	0.1793	0.2881	0.1899	0.1659	0.723	0.966	1.6195	1.4489
	0.0993	0.1542	0.2182	0.17	0.1495	0.938	1.3017	1.2599	1.48
Mean of 6 tech reps	0.10225	0.1649333	0.2063833	0.1700667	0.1659833	0.84975	1.0711833	1.4116167	1.6149667
corrected for media blank		0.0626833	0.1041333	0.0678167	0.0637333	0.7475	0.9689333	1.3093667	1.5127167

Biol Rep 3

		Derm				Mutant			
	Blank	BHIs	0.38% Glu	0.56% Glu	0.9% Glu	BHIs	0.38% Glu	0.56% Glu	0.9% Glu
	0.0886	0.116	0.2132	0.2112	0.2178	0.5349	0.7522	1.1413	1.2967
	0.1062	0.1258	0.1697	0.2695	0.2117	0.6934	0.9069	1.192	1.1597
	0.0807	0.14	0.2285	0.2134	0.2495	0.6598	0.8015	1.0945	1.2128
	0.1016	0.2039	0.1768	0.2755	0.17	0.7765	1.208	1.1435	1.3489
	0.0956	0.1701	0.2063	0.2206	0.2312	0.5229	1.0175	1.1073	1.1032
	0.0829	0.1342	0.2303	0.2513	0.1983	0.5968	0.6697	0.9888	1.5
Mean of 6 tech reps	0.0926	0.1483333	0.2041333	0.24025	0.2130833	0.6307167	0.8926333	1.1112333	1.2702167
corrected for media blank		0.0557333	0.1115333	0.14765	0.1204833	0.5381167	0.8000333	1.0186333	1.1776167

Supplementary Data S4

Collated, averaged, blank-corrected A570 data from above figures

A570 24h

	**Derm**				**Mutant**			
	BHIs	0.38% Glu	0.56% Glu	0.9% Glu	BHIs	0.38% Glu	0.56% Glu	0.9% Glu
Biol Rep 1	0.0236	0.0275	0.021267	0.06435	0.128317	0.634834	0.680867	0.689733
Biol Rep 2	0.034517	0.00945	0.03505	0.042833	0.153233	0.453267	0.48575	0.621567
Biol Rep 3	0.0088	0.011617	0.03345	0.042084	0.107217	0.4533	0.399017	0.41810033
Average	0.022306	0.016189	0.029922	0.049756	0.129589	0.5138	0.521878	0.57646678
SE of mean	0.007452	0.00569	0.004352	0.0073	0.013299	0.060517	0.083344	0.08159166

A570 48h

	**Derm**				**Mutant**			
	BHIs	0.38% Glu	0.56% Glu	0.9% Glu	BHIs	0.38% Glu	0.56% Glu	0.9% Glu
Biol Rep 1	0.037584	0.029367	0.044884	0.1283	0.400867	1.005134	1.3452	1.49983367
Biol Rep 2	0.0286	0.033967	0.046017	0.075217	0.1932	0.731634	1.104784	1.50953367
Biol Rep 3	0.026017	0.025767	0.0937	0.082567	0.240333	1.142483	1.0577	1.0895
Average	0.030734	0.0297	0.061534	0.095361	0.278134	0.95975	1.169228	1.36628911
SE of mean	0.003505	0.002373	0.016087	0.016606	0.062857	0.120753	0.08903	0.13842288

A570 72h

	**Derm**				**Mutant**			
	BHIs	0.38% Glu	0.56% Glu	0.9% Glu	BHIs	0.38% Glu	0.56% Glu	0.9% Glu
Biol Rep 1	0.047467	0.068	0.126134	0.156384	0.573484	1.159484	1.29785	1.54610033
Biol Rep 2	0.062683	0.104133	0.067817	0.063733	0.7475	0.968933	1.309367	1.51271667
Biol Rep 3	0.055733	0.111533	0.14765	0.120483	0.538117	0.800033	1.018633	1.17761667
Average	0.055295	0.094556	0.113867	0.113533	0.6197	0.97615	1.208617	1.41214456
SE of mean	0.004398	0.013448	0.023848	0.026971	0.06471	0.103827	0.09505	0.11765928

Supplementary Data S4

Values used to construct figures and for statistical analysis

A570 Derm — SE of mean

	0 hr	24 hr	48 hr	72 hr	0h	24h	48h	72h
BHIS broth (0.2% glucose)	0	0.022306	0.030734	0.055295	0	0.007452	0.003505	0.004398
0.38% Glucose	0	0.016189	0.0297	0.094556	0	0.00569	0.002373	0.013448
0.56% Glucose	0	0.029922	0.061534	0.113867	0	0.004352	0.016087	0.023848
0.9% Glucose	0	0.049756	0.095361	0.113533	0	0.0073	0.016606	0.026971

A570 Mutant — SE of mean

	0 hr	24 hr	48 hr	72 hr	0h	24h	48h	72h
BHIS broth (0.2% glucose)	0	0.129589	0.278134	0.6197	0	0.013299	0.062857	0.06471
0.38% Glucose	0	0.5138	0.95975	0.97615	0	0.060517	0.120753	0.103827
0.56% Glucose	0	0.521878	1.169228	1.208617	0	0.083344	0.08903	0.09505
0.9% Glucose	0	0.576467	1.366289	1.412145	0	0.081592	0.138423	0.117659

Calculating P values : t-test calculator, available at: http://www.graphpad.com/quickcalcs/ttest1/?Format=SEM

a. Group 1 becomes BHIS values (yellow) and Group 2 becomes 0.9% Glucose values (in green) – Mean values and SEM are in the sheet; N = 3 for each group. Enter data and derive the exact P value.

b. So, for example, for 48 h *Clostridium difficile* 630*Derm*, reported P < 0.05. The exact P value is 0.0190 (statistically significant). Similarly, for 72 h, the exact P value is 0.1001 (not significant).

c. Similarly, for the mutant, the exact P values for 24, 48, and 72 h are 0.0057 (statistically significant), 0.0020 (statistically significant), and 0.0041 (statistically significant), respectively.

Supplementary Data S4

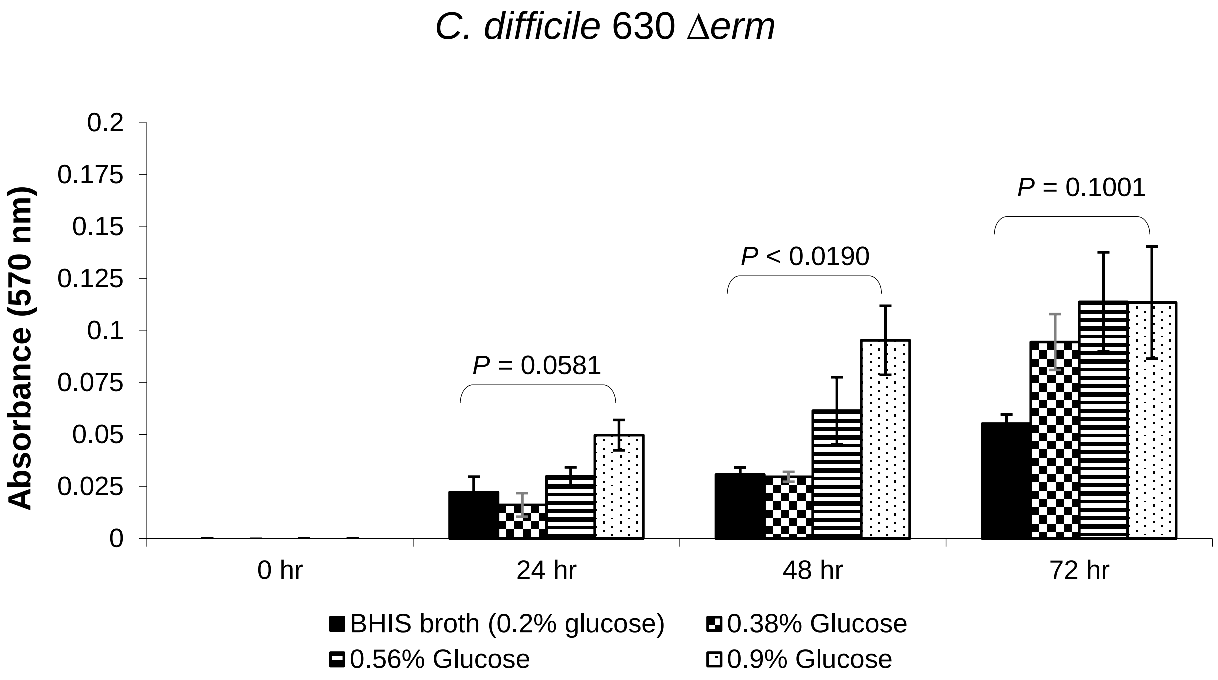

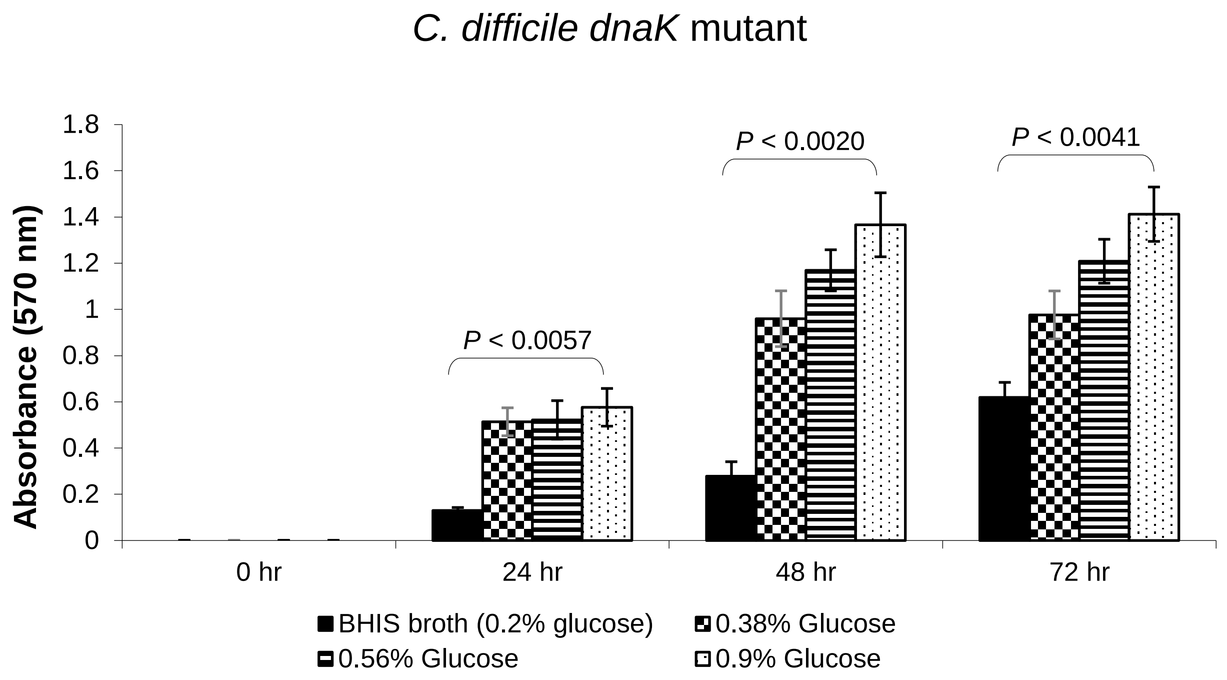

C. difficile dnaK mutant
Absorbance (570 nm)
1.8
1.6
1.4
1.2
1
0.8
0.6
0.4
0.2
0
P < 0.0057
P < 0.0020
P < 0.0041
0 hr
24 hr
48 hr
72 hr
BHIS broth (0.2% glucose)
0.38% Glucose
0.56% Glucose
0.9% Glucose
